# Supplementary material for: Bifidobacteria shape antimicrobial T-helper cell responses during infancy and adulthood
Source: Nat Commun. 2023 Sep 23;14:5943. doi: 10.1038/s41467-023-41630-x (PMC10517955; doi:10.1038/s41467-023-41630-x)
Supplement: Supplementary file 1 — Supplementary Information [file 41467_2023_41630_MOESM1_ESM.pdf]

Supplemental Figures and Tables

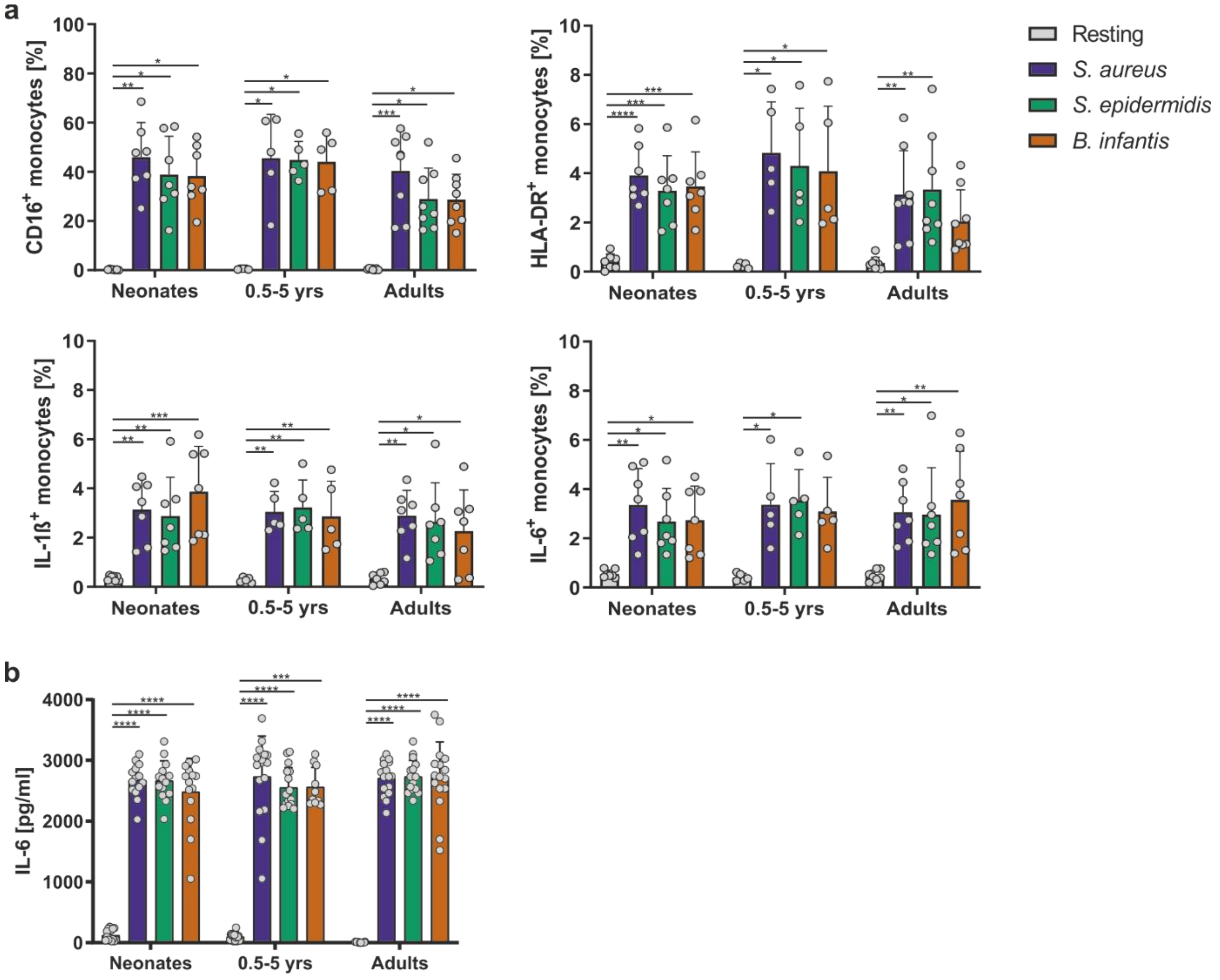

Figure S1. **Maturation of isolated monocytes.** CD14+ monocytes from neonates, children, and adults were cultured with h.i. *S. aureus* (blue bars), h.i. *S. epidermidis* (green bars) and h.i. *B. infantis* (orange bars) for 24 hours. (**a**) Expression of the surface molecules CD16 and HLA-DR as well as percentages of IL-6 and IL-1ß positive cells were measured by flow cytometry. (**b**) Bar graphs showing the IL-6 secretion of bacterially stimulated monocytes from neonates, children, and adults. Error bars in the figures denote mean + SD, n ≥ 5 donors from at least 3 independent experiments in each age group, p-values for HLA-DR expression and IL-1ß expression are calculated by one-way ANOVA followed by Holm-Sidak post hoc test. p-values for CD16 expression, IL-6 expression as well as secretion are calculated by Kruskal Wallis test corrected by Dunn's post hoc test, *p<0.05, **p<0.01, ***p<0.001, ****p<0.0001. Detailed statistical data are provided in Supplementary Table 43-47. Source data are provided as a Source Data file.

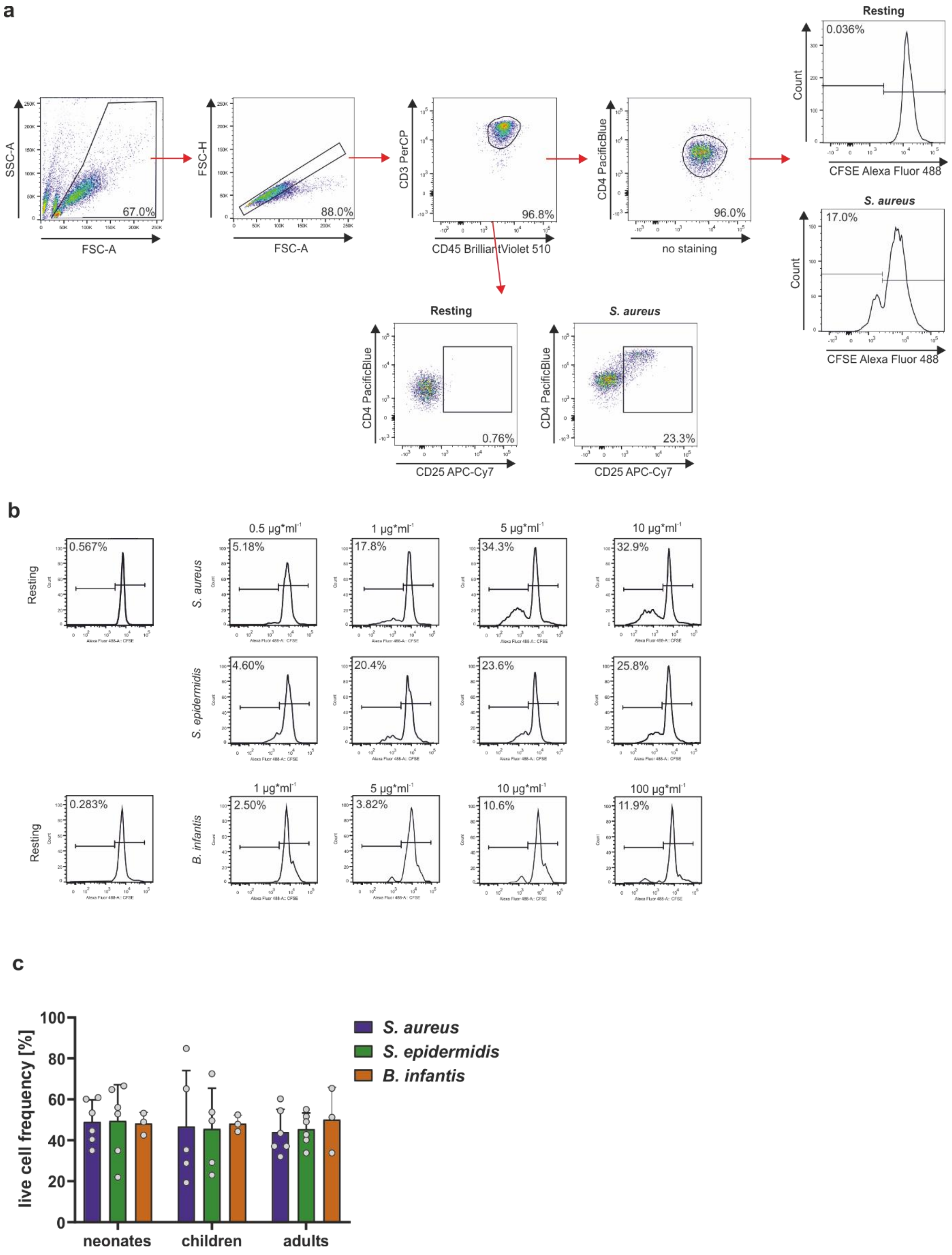
a
SSC-A
FSC-A
67.0%
FSC-H
88.0%
CD3 PerCP
CD45 BrilliantViolet 510
96.8%
CD4 PacificBlue
no staining
96.0%
Resting
0.036%
Count
CFSE Alexa Fluor 488
S. aureus
17.0%
CD25 APC-Cy7
0.76%
23.3%
b
Resting
0.567%
0.5 µg*ml-1
1 µg*ml-1
5 µg*ml-1
10 µg*ml-1
S. aureus
5.18%
17.8%
34.3%
32.9%
S. epidermidis
4.60%
20.4%
23.6%
25.8%
0.283%
100 µg*ml-1
B. infantis
2.50%
3.82%
10.6%
11.9%
c
live cell frequency [%]
neonates
children
adults
S. aureus
S. epidermidis
B. infantis

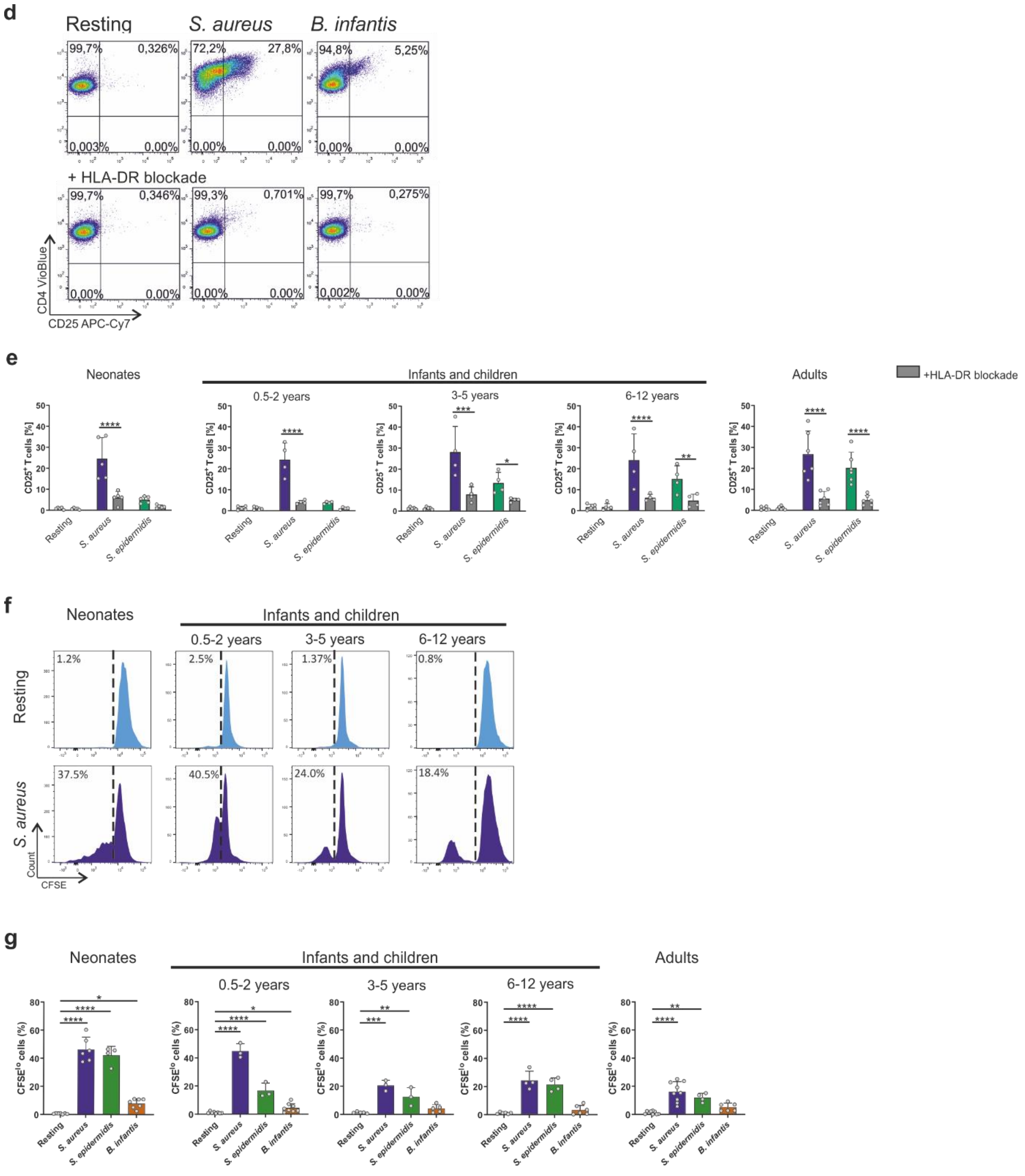

__Figure S2.__ **Bacteria-specific T-cell activation.** (**a**) Gating strategy applied to analyze CD4^{+} T-cells. (**b**) Concentrations of h.i. bacteria for experiments were determined by identifying max. T-cell proliferation upon *in vitro* priming of human naïve T-cells with bacteria-matured monocytes as indicated. (**c**) Analysis of T-cell survival**.** T-cells from neonates, children, and adults were co-cultured with monocytes matured with h.i. *S. aureus* (blue bars), h.i. *S. epidermidis* (green bars) and h.i. *B. infantis* (orange bars) for 6 days. Bar graph showing life cells identified as propidium iodide and annexin V negative by flow cytometry. Cumulative results are shown and each dot represents a

different donor. Error bars in the figures denote ± SD. (**d**) Representative dot plots from a 2-year-old boy showing the frequency of naïve T-cells expressing CD25 after 3 days of stimulation with monocytes matured with h.i. *S. aureus* or *B. infantis* in the presence or absence of HLA-DR blocking antibody. Data are representative for at least five donors from three experiments. (**e**) Bar graph showing CD25 expression of naïve T-cells isolated form neonates, infants, children and adults in response to h.i. *S. aureus*- (blue) or h.i. *S. epidermidis*-antigen (green) in the presence or absence of HLA-DR blocking antibody for 3 days as determined by flow cytometry. (**f**) Purified naïve ($CD4^{+}CD45RA^{+}CD31^{+}$) T-cells were labelled with CSFE and co-cultured at a ratio of 2:1 with monocytes matured with h.i. *S. aureus*-antigen. CFSE dilution profiles and frequencies of proliferating ($CFSE^{lo}$) T- cells from neonates, infants and children were measured on day 3 after stimulation by flow cytometry. Data are representative for at least five donors from three experiments. (**g**) Bar graphs showing frequency of $CFSE^{lo}$ T-cells upon stimulation with monocyte (as shown in Fig. S1) matured h.i. *S. aureus*- (blue), h.i. *S. epidermidis*- (green) or h.i. *B. infantis*-antigen (orange) for 3 days. Error bars in the figures (**c, e**) and (**g**) indicate mean + SD, $n \geq 4$ donors from at least 2 independent experiments in each age group, p-values in (**c, e**) and (**g**) are calculated by one-way ANOVA followed by Holm-Sidak post hoc test, *p<0.05, **p<0.01, ***p<0.001, ****p<0.0001. Detailed statistical data are provided in Supplementary Table 48-50. Source data are provided as a Source Data file.

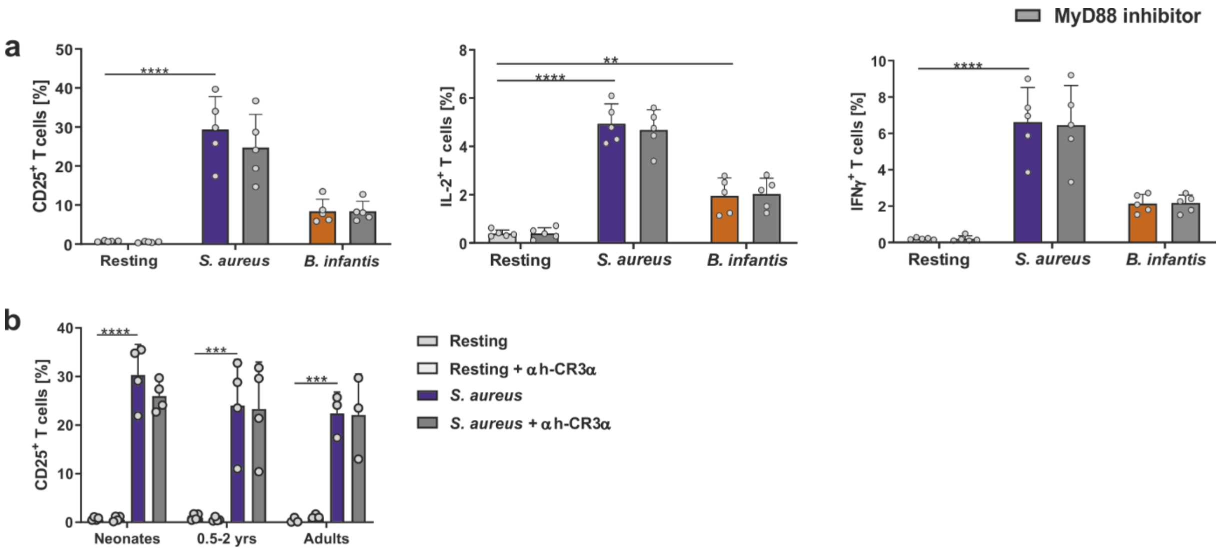

Figure S3. **T-cell activation is TLR-independent.** (**a**) Adult $CD4^+CD45RA^+CD31^+$ T-cells were co-cultured with monocytes matured with h.i. *S. aureus*- or h.i. *B. infantis*-lysates in the presence or absence of the MyD88-inhibitor Pepinh-MYD. Surface expression of CD25 as well as intracellular cytokine expression of IL-2 and IFNγ were measured by flow cytometry and the frequencies of these cells from adults are presented as bar graphs. (**b**) The frequency of T-cells from neonates, infants and adults expressing CD25 in the presence or absence of anti-human CR3α upon 3 days after stimulation with monocytes matured with h.i. *S. aureus* was measured by flow cytometry. Cumulative results are shown and each dot represents a different donor. Error bars in the figures indicate mean + SD, $n \geq 4$ donors from at least 2 independent experiments in each age group, p-values are calculated by one-way ANOVA followed by Holm-Sidak post hoc test, *p<0.05, **p<0.01, ***p<0.001, ****p<0.0001. Detailed statistical data are provided in Supplementary Table 51-54. Source data are provided as a Source Data file.

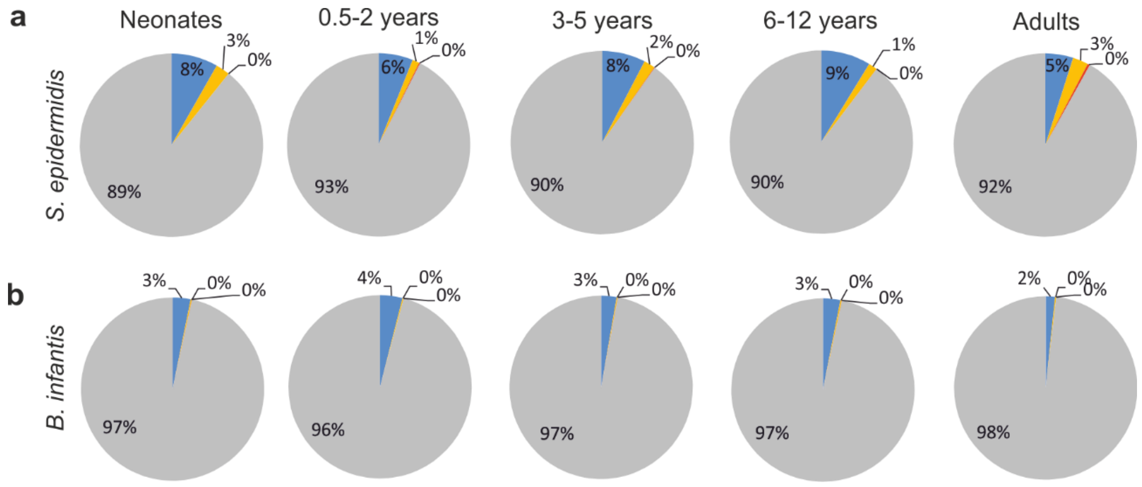

Figure S4. **Age-dependent formation of multi-functional Th1 cells.** $CD4^{+}CD45RA^{+}CD31^{+}$ T-cells were stimulated with h.i. *S. epidermidis* (**a**) or h.i. *B. infantis* (**b**) and the cells expressing single or multiple cytokines in respect of IL-2, TNF-α, and IFN-γ were determined by flow cytometry, analysed by Boolean gating and shown as a fraction of all $CD4^{+}$ T-cells in a pie chart. The subsets expressing no (grey), one (blue), two (yellow) or three (red) different cytokines simultaneously are grouped by colour. Cumulative results from five donors are shown from three independent experiments. Detailed statistical data are provided in Supplementary Table 55 and 56. Source data are provided as a Source Data file.

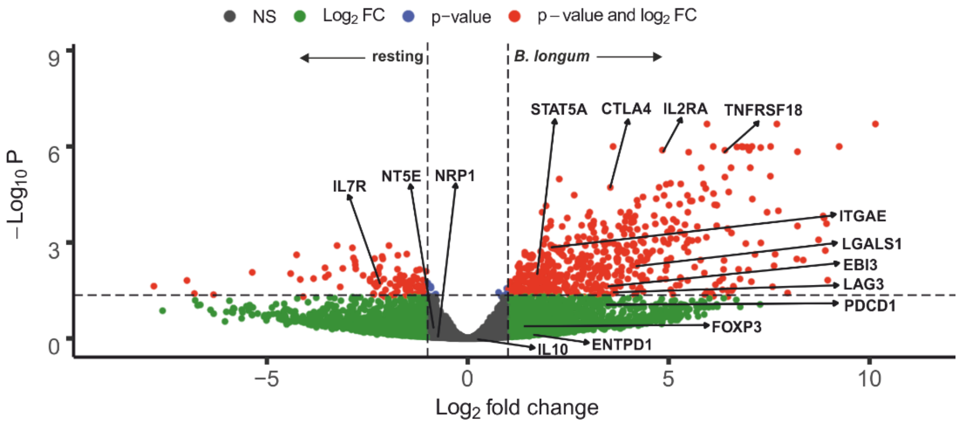

Figure S5. **Primary *B. infantis*-stimulated T-cells from adult show a regulatory RNA pattern.** $CD4^+CD45RA^+CD31^+$ T-cells from adults were stimulated for 3 days with monocytes matured with h.i. *B. infantis* (as in Fig. 1). T-cells were enriched using MACSQuantTyto, RNA was prepared and RNASeq was performed as described in Materials and Methods. Volcano plot of differentially expressed mRNA: Green and red dots indicate differentially regulated genes. Data are representative for four donors from four independent experiments.

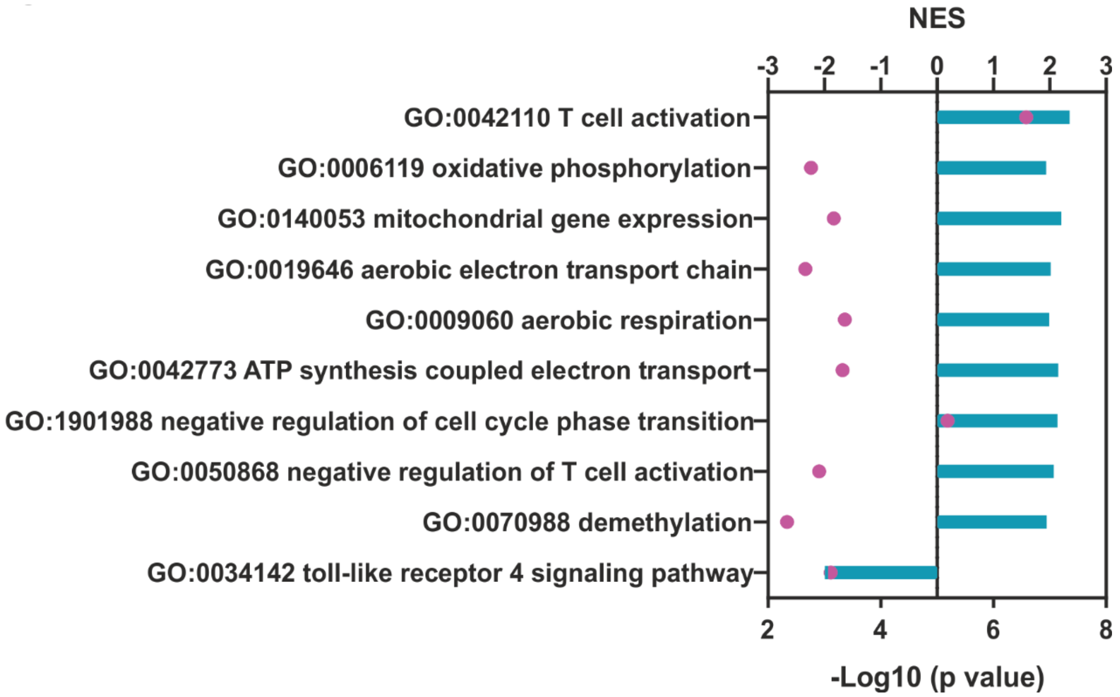

<u>Figure S6.</u> **Gene ontology (GO) enrichment analysis of differentially expressed genes in *B. infantis*-stimulated neonatal T-cells.** Naïve T-cells from neonates were co-cultured with monocytes matured with h.i. *B. infantis* for 3 days. T-cells were enriched using MACSQuantTyto, RNA was prepared and RNASeq was performed as described in Materials and Methods. Blue bars indicate normalized enrichment scores (NES) of GO categories. Pink dots indicate p-value calculated for each GO category. Data are representative of four donors from four independent experiments.

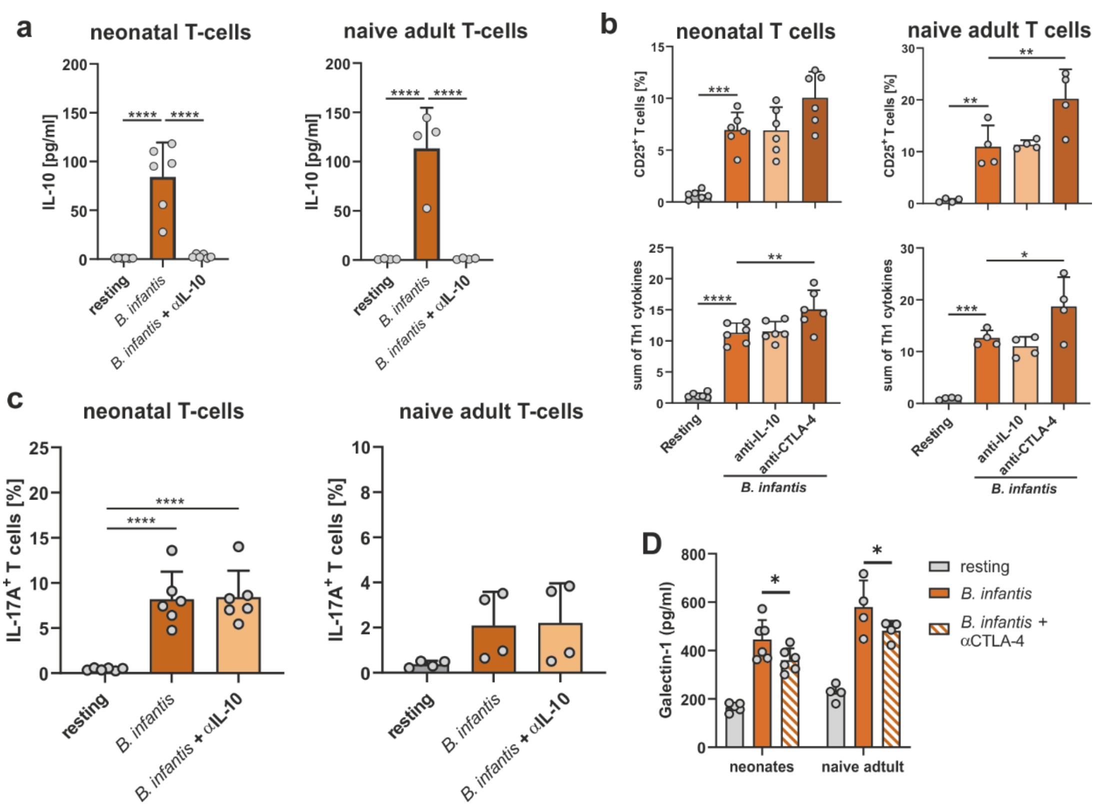

Figure S7. **Role of CTLA-4 and IL-10 in the *B. infantis*-specific T-cell response.** Naïve T-cells from neonates or adults were co-cultured with monocytes matured with h.i. *B. infantis* in the presence or absence of specific antibodies against IL-10 or CTLA-4 as indicated. (**a**) $CD4^{+}CD45RA^{+}CD31^{+}$ T-cells were co-cultured in the presence or absence of specific antibody blocking IL-10. Determination of IL-10 cytokine release from neonatal (left) as well as adult naïve T-cells (right) measured by LEGENDplex. (**b**) Bar graph showing the frequency of CD25 expression (upper panel) and the sum of T-cells expressing IL-2, TNF-α, and IFN-γ (lower panel) from neonates (left) as well as naïve T-cells from adults (right) as determined by flow cytometry. (**c**) Bar graphs showing frequency of IL-17A cytokine producers of neonatal (left) as well as adult naïve T-cells (right) measured by flow cytometry. (**d**) Bar graph showing the level of galectin-1 release from T-cells of neonates and adults after 3 days of stimulation, as measured by ELISA assay. Cumulative results are shown, and each dot represents a different donor. Error bars in the figures indicate mean + SD, n ≥ 4 donors from at least 2 independent experiments in each age group, p-values are calculated by one-way ANOVA followed by Holm-Sidak post hoc test, *p<0.05, **p<0.01, ***p<0.001, ****p<0.0001. Detailed statistical data are provided in Supplementary Table 57-64. Source data are provided as a Source Data file.

Table S1. Characteristics of neonatal research subjects and their mothers.		
**Characteristics**	**Neonates (n=23)**	**Mothers (n=23)**
**Gestational age – week of gestation (range)**	39.3±1.3 (37-41)	
**Sex – no. (%)**		
**Male**	13 (56.5)	
**Female**	9 (39.1)	
**Height – cm**	51.9±2.8	
**Weight – cm**	3596±473	
**Caesarean section – no. (%)**	6 (26.1)	
**Medicinal induction of labour – no. (%)**	5 (21.7)	
**Age – y. (range)**		30±4 (22-37)
**Allergy – no. (%)**		11 (47.8)
**Medication taken during pregnancy – no. (%)**		7 (30.4)
**Co-morbidities such as – no. (%)**		
**Disease of the nervous system**		2 (8.7)
**Metabolic disorder**		3 (13)
**Blood clotting disorder**		1 (4.35)
**Drug allergy**		2 (8.7)
**Acute infection**		1 (4.35)
**Disease of the gastrointestinal tract**		1 (4.35)

The size of each group is indicated in total numbers (no.) as well as percentages (%) in respect to the entire collective. For one neonatal research, subject information about gender was not available.

Table S2. Characteristics of child research subjects.			
**Characteristics**	**Children 0.5-2 years (n=14)**	**Children 3-5 years (n=12)**	**Children 6-12 years (n=9)**
**Age – y.**	1.73±0.35	3.28±0.52	6.45±1.23
**Sex – no. (%)**			
**Male**	9 (64.3)	6 (50)	5 (55.6)
**Female**	5 (35.7)	6 (50)	4 (44.4)
**Brest feeding – no. (%)**	4 (28.6)	5 (41.7)	3 (33.3)
**Allergy – no. (%)**	1 (7.2)	1 (8.3)	0
**Antibiotics within the first 2 years of life – no. (%)**	9 (64.3)	4 (33.3)	3 (33.3)
**Nasal obstruction such as – no. (%)**			
**Clinical nasal obstruction**	11 (78.6)	9 (75)	6 (66.6)
**Snoring**	12 (85.7)	10 (83.3)	9 (100)
**“Nasal” pronunciation**	5 (35.7)	7 (58.3)	7 (77.7)
**Upper airway infections such as – no. (%)**			
**Recurrent rhinitis/ pharyngitis**	10 (71.4)	7 (58.3)	6 (66.6)
**Recurrent bronchitis**	7 (50)	4 (33.3)	3 (33.3)
**Otitis media with effusion such as – no. (%)**			
**Recurrent otitis**	6 (42.9)	1 (8.3)	2 (22.2)
**Hearing impairment**	6 (42.9)	5 (41.7)	7 (77.7)
**Acute infection – no. (%)**	0	0	0

The size of each group is indicated in total numbers (no.) as well as percentages (%) in respect to the entire collective.

Table S3. Characteristics of adult research subjects.		
**Characteristics**	**Healthy Adults (n=24)**	**Acute, severe COVID-19 patients (n=5)**
**Age – y. (range)**	44±11 (20-59)	61±9 (45-72)
**Sex – no. (%)**		
**Male**	17 (70.8)	2 (40)
**Female**	7 (29.2)	3 (60)
**COVID-19 severity**		
**Mechanical ventilation – no. (%)**	0	4 (80)
**Extracorporeal membrane oxygenation (ECMO) support – no. (%)**	0	2 (40)
**Simplified Acute Physiology Score (SAPS) II (range)**	0	39±13 (20-53)
**Personal history of autoimmune diseases – no. (%)**	0	1 (20)
**Co-morbidities such as – no. (%)**		
**Hypertension**	NA	5 (100)
**Diabetes**	NA	1 (20)
**Airway disease**	NA	1 (20)
**Thyroid disease**	NA	1 (20)
**Obesity**	NA	1 (20)
**Disease of the nervous system**	0	3 (60)

The size of each group is indicated in total numbers (no.) as well as percentages (%) in respect to the entire collective. NA: not available

Table S4. Key Resources Table of the antibodies, bacterial strains, reagents, culture media, buffers and software used.		
**Reagent or Resource**	**Source**	**Identifier**
**Antibodies**		
CD14 – VioBlue (clone: REA599)	Miltenyi Biotec	Order no: 130-110-524
CD16 – APC-Vio770 (clone: REA423)	Miltenyi Biotec	Order no: 130-106-707
CD45RA – FITC (clone: REA562)	Miltenyi Biotec	Order no: 130-113-927
CD45RO – PE-Vio770 (clone: REA611)	Miltenyi Biotec	Order no: 130-113-560
CD4 - FITC (clone: REA613)	Miltenyi Biotec	Order no: 130-114-531
CD3 - VioGreen (clone: REA623)	Miltenyi Biotec	Order no: 130-113-142
CD14 – PerCP-Vio700 (clone: REA599)	Miltenyi Biotec	Order no:130-110-523
CD4 –PacificBlue (clone: RPA-T4)	Biolegend	Cat:300521
CD3 – PerCP (clone: SK7)	Biolegend	Cat: 344814
CD25 – APC-Cy7 (clone:BC96)	Biolegend	Cat: 302614
CD45 – Brilliant Violet 510 (clone: HI30)	Biolegend	Cat:304036
IL-2 -APC (clone: MQ1-17H12)	Biolegend	Cat:500311
TNF-α-FITC (clone: Mab11)	Biolegend	Cat:502906
CD69 – FITC (clone: FN50)	Biolegend	Cat: 310904
anti-T-bet - PE-Cy7 (clone: 4B10)	Biolegend	Cat:644824
anti-FoxP3 – Alexa Fluor 647(clone: 259D)	Biolegend	Cat:320214
anti-GATA3 – Alexa Fluor 488 (clone: 16E10A23)	BioLegend	Cat:653808
Ultra-LEAF Purified IL-10 (clone: JES3-19F1)	Biolegend	Cat: 506814
LEAF Purified CD11b (clone: ICRF44)	Biolegend	Cat:301312
LEAF Purified CD18 (clone: TS1/18)	Biolegend	Cat:302112
LEAF Purified TGFß (clone: 19D8)	Biolegend	Cat:521704
Ultra-LEAF Purified CD3 (clone:UCHT1)	Biolegend	Cat:300438
Ultra-LEAF Purified CD28 (clone: CD28.2)	Biolegend	Cat:302934
IL-17A-PE (clone: eBio64DEC17)	eBioscience	Cat #12-7179-42
IFN-γ –PE-Cy7 (clone: 4S.B3)	BD Biosciences	Cat:557844
RORγt – PE (clone: Q21-559)	BD Biosciences	Cat: 563081
IL-10 – APC (clone: JES3-19F1)	BD Biosciences	Cat:562036
CTLA-4 (clone: BNI3)	BD Pharmingen	Cat:550405
HLA-DR – PE-Cy7 (clone:L243)	Biolegend	Cat:307615
IL-6 – APC (clone:MQ2-13A5)	Biolegend	Cat:501112
IL-1ß – PE (clone: CRM56)	Invitrogen	Cat#12-7018-81
Annexin V – APC	Biolegend	Cat:640941

**Bacterial Strains**		
Bifidobacterium longum ssp. infantis	ATCC	Cat# ATCC-15697
Staphylococcus aureus ssp. aureus	ATCC	Cat# ATCC-25923
Staphylococcus epidermidis	ATCC	Cat# ATCC-12228
**Reagents, Culture Media and Buffer**		
BD Horizon Brilliant Stain Buffer	BD Biosciences	Cat:563794
FcR Blocking Reagent	Miltenyi Biotec	Order no: 130-059-901
DPBS – Dulbeccos Phosphate Buffered Saline	Life Technologies GmbH	Cat:14190169
RPMI 1640	PAN Biotech	Cat: P04-18500
Albumin Fraktion V, endotoxin-checked <15EU	ROTH	Art.-Nr.CP84-2
Penicillin/ Streptomycin	Life Technologies GmbH	Cat:15140122
Fetal Bovine Serum	Gibco/Life Technologies GmbH	Cat:10270-106
Pancoll human	PAN Biotech	Cat: P04-60500
Formaldehyde solution 37%	Merck	Cat:8.18708.1000
Methanol	Carl Roth	Art.-Nr.8388.1
PMA	Sigma-Aldrich	Cat:P8139
Ionomycin	Cell Signaling	Cat: 9995 S
Brefeldin A	Cell Signaling	Cat: 9972
PepTivator SARS-CoV-2 Prot_S	Miltenyi Biotec	Order no: 130-129-928
PepTivator SARS-CoV-2 Prot_N	Miltenyi Biotec	Order no: 130-126-698
MyD88 inhibitor Pepinh-MYD	InvivoGen	Cat: tlrl-pimyd
Paraformaldehyde	Morphisto GmbH	Cat: 11762.01000
Saponin	Sigma-Aldrich	Cat:S7900-100G
CFSE	Molecular Probes	Cat:C1157
Propidium Iodid	Biolegend	Cat: 421301
MACSQuant Tyto Running Buffer	Miltenyi Biotec	Order no: 130-107-207
**Commercial Assays**		
CD14 MicroBeads human	Miltenyi Biotec	Order no: 130-050-201
naive CD4+ T-cell Isolation Kit human	Miltenyi Biotec	Order no: 130-094-131
human CD4+ Recent Thymic Emigrant Isolation Kit	Miltenyi Biotec	Order no: 130-093-227
LEGENDplex human Th Cytokine Panel (12 plex)	Biolegend	Cat: 741027
Pierce LAL Chromogenic	Thermo Scientific	Cat: A39552

Endotoxin Quantitation Kit		
Human IFN-γ/IL-10 Double-Color Enzymatic ELISPOT Assay	CTL-Europe GmbH	Cat: #hIFNgIL10-2M/2
NucleoSpin RNA isolation kit	Macherey-Nagel	Cat: 740955.50
Galectin-1 ELISA	R&D Systems	Cat: #DGAL10
Collibri 3´ mRNA Library Prep Kit	Invitrogen	Cat: A38110096
NextSeq 500/550 High Output Kit v2.5 (75 Cycles)	Illumina	Cat: 20024906
**Software**		
FlowJo (version 10.8.1)	Treestar	www.flowjo.com
LEGENDplex Data Analysis Software Suite	Qognit	https://legendplex.qognit.com/user/login?next=home
FACS Diva software (version 9.0.1)	BD Bioscinces	www.bdbiosciences.com
FASTQC tool	Babraham Institute	https://www.bioinformatics.babraham.ac.uk/projects/fastqc/
Trim Galore! wrapper tool	Babraham Institute	https://www.bioinformatics.babraham.ac.uk/projects/trim_galore/
short read aligner STAR		https://code.google.com/p/rna-star/
R package "Rsubread"	Bioconductor	https://bioconductor.org/packages/release/bioc/html/Rsubread.html
R package "bioMaRt	Bioconductor	https://bioconductor.org/packages/release/bioc/html/biomaRt.html
R package "edgeR"	Bioconductor	https://bioconductor.org/packages/release/bioc/html/edgeR.html
R package "clusterProfiler"	Bioconductor	https://bioconductor.org/packages/release/bioc/html/clusterProfiler.html
GraphPad Prism 9	GraphPad	www.graphpad.com
Jamovi (version 2.2.21)	Jamovi	www.jamovi.org

Identified key resources of the antibodies, bacterial strains, reagents, culture media, buffers and software used. Further information and requests for reagents or key resources could be directed to, and will be fulfilled by Katrin Vogel (katrin.vogel@med.ovgu.de).

Table S5. Summarized data of cell clusters formed.													
**Study (Corres-ponding figure)**	**Condition**	**Mean**	**SD**	***n***	**Mean**	**SD**	***n***	***P* Value**			***P* Value**		
		**neonatal T-cells**			**naïve adult T-cells**			**neonatal T-cells**			**naïve adult T-cells**		
								Rest.	*S. aureus*	*B. infantis*	Rest.	*S. aureus*	*B. infantis*
No. of clusters	only T-cells	0.333	0.577	3	0.667	0.577	3						
(Fig. 1C)	Resting	5.667	2.082	3	6.667	2.517	3						
	S. aureus	114.33	22.23	3	119.33	12.01	3	0.0001			<0.0001		
	B. infantis	120.34	29.57	3	113.41	1.528	3	<0.0001	0.8955		<0.0001	0.4423	
	αCD3/αCD28	96.00	9.54	3	104.67	5.508	3	0.0004	0.5233	0.3788	<0.0001	0.5561	0.2961

Rest.-Resting, n-number of donors, p values obtained from one-way ANOVA followed by Holm-Sidak post hoc test

Table S6. Extensive statistical data of cytokine expression and secretion by *S. aureus* and *B. infantis* in addition to Fig. 2A and B.

Study (Corresponding figure)	Age group	Mean	SD	*n*	Mean	SD	*n*	Mean	SD	*n*	*P* Value	*P* Value				*P* Value	*P* Value			
		**resting**			***S. aureus***			***B. infantis***			Rest vs. *S. aureus*	age vs. *S. aureus*				Rest vs. *B. infantis*	age vs. *B. infantis*			
												Neo	0.5-2	3-5	6-12		Neo	0.5-2	3-5	6-12
% of IL-2^+ T-ce	Neonates	0.403	0.242	5	5.270	2.120	5	0.973	0.298	5	0.0039					0.0136				
(Fig. 2A,left)	0.5-2 yrs.	0.253	0.142	6	5.338	2.811	6	0.838	0.212	6	0.0007	0.9676				0.0044	0.7948			
	3-5 yrs.	0.348	0.236	6	4.162	3.226	6	1.207	0.334	6	0.0185	0.8946	0.8930			<0.0001	0.4926	0.1385		
	6-12 yrs.	0.193	0.131	5	6.854	4.251	5	0.908	0.550	5	<0.0001	0.8549	0.8549	0.2631		0.0014	0.8908	0.8908	0.3309	
	Adults	0.135	0.133	8	4.408	1.491	8	0.388	0.228	8	0.0011	0.8946	0.8946	0.9676	0.2811	0.3309	0.0044	0.0250	<0.0001	0.0135
% of TNFα$^+$ T-c	Neonates	0.459	0.351	7	22. Jan	7.590	7	1.522	0.555	7	<0.0001					0.0368				
(Fig. 2A, midd	0.5-2 yrs.	0.343	0.228	6	9.258	3.776	6	1.473	0.578	6	0.0016	<0.0001				0.0210	>0.9999			
	3-5 yrs.	0.341	0.262	6	5.942	4.643	6	0.693	0.410	6	0.0831	<0.0001	0.3639			>0.9999	0.8284	>0.9999		
	6-12 yrs.	0.404	0.382	5	Okt 53	7.403	6	0.594	0.386	6	0.0011	<0.0001	0.5856	0.2391		>0.9999	0.3556	0.5214	>0.9999	
	Adults	0.404	0.285	8	3.596	2.604	8	0.451	0.254	8	0.3486	<0.0001	0.0576	0.4557	0.0197	>0.9999	0.0244	0.0502	>0.9999	>0.9999
% of IFNγ$^+$ T-ce	Neonates	0.161	0.147	5	3.348	1.049	5	0.229	0.103	5	0.0008					0.8691				
(Fig. 2A, right	0.5-2 yrs.	0.164	0.152	6	3.528	0.7446	6	1.069	0.258	6	0.0001	0.9244				<0.0001	<0.0001			
	3-5 yrs.	0.308	0.139	5	3.850	0.7676	5	1.071	0.323	5	0.0002	0.9244	0.9244			<0.0001	<0.0001	0.9852		
	6-12 yrs.	0.089	0.081	5	5.374	2.363	5	1.336	0.231	5	<0.0001	0.0772	0.0975	0.2551		<0.0001	<0.0001	0.3061	0.3151	
	Adults	0.337	0.288	6	4.872	2.277	6	0.868	0.329	6	<0.0001	0.2447	0.2638	0.5476	0.9244	0.0017	0.0003	0.4377	0.4377	0.0103
Secreted IL-2 (	Neonates	3.360	1.672	8	189.4	62.88	8	31.25	9.739	8	<0.0001					<0.0001				
(Fig. 2B, left)	0.5-2 yrs.	2.713	0.996	6	371.4	153.9	6	26. Feb	5.851	6	<0.0001	0.0162				<0.0001	0.5169			
	3-5 yrs.	2.098	0.922	6	342.5	154.9	6	25.67	5.220	6	<0.0001	0.0543	0.7335			<0.0001	0.5169	0.9996		
	6-12 yrs.	2.433	1.079	6	227.5	38.19	6	25.74	6.012	6	<0.0001	0.7335	0.1036	0.2010		<0.0001	0.5169	0.9996	0.9996	
	Adults	2.193	0.573	8	113.1	44.07	8	3.895	1.520	8	0.0025	0.3553	0.0004	0.0017	0.1927	0.4581	<0.0001	<0.0001	<0.0001	<0.0001
Secreted TNFα	Neonates	3.975	2.434	8	540.5	170.7	8	234.8	107.6	8	<0.0001					<0.0001				
(Fig. 2B, midd	0.5-2 yrs.	2.913	1.347	6	137.7	23.88	6	30.56	Dez 16	6	0.0289	0.0073				0.0276	0.0090			
	3-5 yrs.	2.442	0.301	6	140.2	21.13	6	30.14	Dez 43	6	0.0125	0.0082	>0.9999			0.0151	0.0057	>0.9999		
	6-12 yrs.	3.853	0.641	6	177.0	45.38	6	33.60	13.89	6	0.0737	0.2566	>0.9999	>0.9999		0.2602	0.0112	>0.9999	>0.9999	
	Adults	3.079	13.89	8	131.9	85.83	8	43.36	23.77	8	0.0125	0.0012	>0.9999	>0.9999	>0.9999	0.0022	0.0446	>0.9999	>0.9999	>0.9999
Secreted IFNγ	Neonates	2.675	1.131	8	178.4	26.15	8	16.86	6.357	8	<0.0001					0.0052				
(Fig. 2B, right	0.5-2 yrs.	4.002	1.089	6	168.8	48.77	6	42.42	Nov 14	6	<0.0001	0.9579				<0.0001	0.0121			
	3-5 yrs.	4.208	1.144	6	124.4	34.81	6	38.12	Okt 28	6	0.0003	0.6017	0.6957			<0.0001	0.0373	0.5918		
	6-12 yrs.	5.645	2.034	6	402.9	59.99	6	69.14	16.53	6	<0.0001	<0.0001	<0.0001	<0.0001		<0.0001	<0.0001	0.0126	0.0040	
	Adults	3.708	1.999	8	392.0	137.3	8	51.50	19.48	8	<0.0001	<0.0001	<0.0001	<0.0001	0.9579	<0.0001	0.0002	0.4067	0.2249	0.0927

Rest.-Resting, Neo-Neonates, n-number of donors, p values obtained from Kruskal Wallis test followed by Dunn's post hoc test.

**Table S7. Summarized statistical data of T-bet expression in addition to Fig. 2C**																			
**Study (Corres-ponding figure)**	**Age group**	**Mean**	**SD**	***n***	**Mean**	**SD**	***n***	**Mean**	**SD**	***n***	**Mean**	**SD**	***n***	***P*** **Value**		***P Value***		***P*** **Value**	
		**resting**			***S. aureus***			***S. epidermidis***			***B. infantis***			***S. aureus***		***S. epidermidis***		***B. infantis***	
														Rest. vs. *S. aureus*	age vs. *S. aureus*	Rest. vs. *S. epidermidis*	age vs. *S. epidermidis*	Rest. vs. *B.infantis*	age vs. *B. infantis*
% of T-bet⁺ T-cells	Neonates	0.587	0.259	6	7.313	2.999	6	5.777	1.190	6	3.782	2.251	6	<0.0001		0.0016		0.0229	
(Fig. 2C)	Adults	0.481	0.183	9	6.402	4.103	9	5.327	2.134	5	3.948	1.704	5	<0.0001	0.8472	0.0018	0.9392	0.0223	0.9392

Rest.-Resting, n-number of donors, p values obtained from Kruskal Wallis test followed by Dunn's post hoc test

**Table S8. Detailed statistical data of IFNγ expression by *S. epidermidis* in addition to Fig. 2D.**												
**Study (Corresponding figure)**	**Age group**	**Mean**	**SD**	***n***	**Mean**	**SD**	***n***	***P*** **Value**	***P*** **Value**			
		**resting**			***S. epidermidis***			Rest. vs. *S. epidermidis*	age vs. *S. epidermidis*			
									Neo	0.5-2	3-5	6-12
% of IFNγ⁺ T-cells	Neonates	0.161	0.147	5	1.762	0.267	5	0.0137				
(Fig. 2D)	0.5-2 yrs.	0.164	0.152	6	1.281	0.440	6	0.0693	0.7609			
	3-5 yrs.	0.308	0.139	5	3.218	1.411	5	<0.0001	0.0278	0.0012		
	6-12 yrs.	0.089	0.081	5	3.375	1.363	5	<0.0001	0.0111	0.0004	0.9203	
	Adults	0.337	0.288	6	3.155	1.104	6	<0.0001	0.0278	0.0011	0.9203	0.9203

Rest.-Resting, Neo-Neonates, n-number of donors, p values obtained from Kruskal Wallis test followed by Dunn's post hoc test

**Table S9. Supplementary statistical data to Fig. 2E about the influence of MyD88 inhibition on T cell activation.**														
**Study (Corresponding figure)**	**Condition**	**Mean**	**SD**	***n***	**Mean**	**SD**	***n***	**Mean**	**SD**	***n***	***P* Value**		***P* Value**	
		**resting**			***S. aureus***			***B. infantis***			***S. aureus***		***B. infantis***	
											Rest. vs. *S. aureus*	*S. aureus* Ø MyD88-Inhibitor vs. *S. aureus* + MyD88-Inhibitor	Rest. vs. *B.infantis*	*B. infantis* Ø MyD88-Inhibitor vs. *B. infantis* + MyD88-Inhibitor
% of TNFα^{+} T-cells	Ø MyD88-Inhibitor	0.336	0.090	5	6.206	0.7013	5	2.276	1.126	5	<0.0001		0.0112	
(Fig.2E)	+ MyD88-Inhibitor	0.313	0.061	5	5.714	1.125	5	1.989	1.003	5	<0.0001	0.6376	0.0263	0.6376

Rest.-Resting, n-number of donors, p values obtained from Kruskal Wallis test followed by Dunn's post hoc test

Table S10. Summarized statistical data of multiple cytokine producers by *S. aureus* in addition to Fig. 2F.																	
**Study (Corresponding figure)**	**Age group**	**Mean**	**SD**	***n***	**Mean**	**SD**	***n***	**Mean**	**SD**	***n***	**Mean**	**SD**	***n***	***P* Value**			
		Single producers			***Double producers***			***Triple producers***			***Non producers***			age vs. *S. aureus*			
														Neo	0.5-2	3-5	6-12
% of cytokine	Neonates	19.77	15.74	8	12.31	5.019	8	2.69	2.546	8	65.15	24.55	8				
producing T-cells	0.5-2 yrs.	14.72	4.940	6	5.35	2.797	6	4.15	8.441	6	76.92	19.98	6	0.1895			
(Fig. 2F)	3-5 yrs.	8.78	3.280	6	4.42	2.587	6	4.17	8.454	6	82.62	20.38	6	0.0097	0.6099		
	6-12 yrs.	11.63	5.949	5	4.41	4.660	5	1.17	2.861	5	82.79	18.53	5	0.0198	0.5230	0.6138	
	Adults	6.91	1.960	8	1.03	0.4234	8	1.08	0.9256	8	91.22	4.674	8	<0.0001	0.0279	0.2613	0.2544

Rest.-Resting, Neo-Neonates, n-number of donors, p values obtained from Fisher's Exact Test

Table S11. Detailed statistical data of IL-17A producers by *S. aureus* and *B. infantis* in addition to Fig. 2G (left bar graph).																				
**Study (Corresponding figure)**	**Age group**	**Mean**	**SD**	***n***	**Mean**	**SD**	***n***	**Mean**	**SD**	***n***	***P* Value**	***P* Value**				***P* Value**	***P* Value**			
		**resting**			***S. aureus***			***B. infantis***			Rest vs. *S. aureus*	age vs. *S. aureus*				Rest. vs. *B. infantis*	age vs. *B. infantis*			
												Neo	0.5-2	3-5	6-12		Neo	0.5-2	3-5	6-12
% of IL-17A$^+$ T-cells	Neonates	0.495	0.303	6	3.705	2.063	6	0.735	0.783	6	<0.0001					0.9792				
(Fig. 2G left)	0.5-2 yrs.	0.570	0.395	5	3.518	1.563	5	0.798	0.686	5	0.0001	0.9337				0.9847	0.9847			
	3-5 yrs.	0.554	0.238	5	2.342	0.336	5	1.008	0.174	5	0.0330	0.1341	0.2784			0.8432	0.9779	0.9874		
	6-12 yrs.	0.312	0.166	5	1.292	0.923	5	0.902	0.689	5	0.3566	0.0012	0.0050	0.3501		0.5714	0.9874	0.9874	0.9874	
	Adults	0.282	0.186	5	0.314	0.188	5	0.444	0.332	5	0.9586	<0.0001	<0.0001	0.0120	0.3566	0.9847	0.9772	0.9531	0.6156	0.8432

Rest.-Resting, Neo-Neonates, n-number of donors, p values obtained from Kruskal Wallis test followed by Dunn's post hoc test

**Table S12. Summarized statistical data of IL-17A producers by *S. epidermidis* in addition to Fig. 2G (right bar graph).**												
**Study (Corresponding figure)**	**Age group**	**Mean**	**SD**	***n***	**Mean**	**SD**	***n***	***P* Value**	***P* Value**			
		**resting**			***S. epidermidis***			Rest. vs. *S. epidermidis*	age vs. *S. epidermidis*			
									Neo	0.5-2	3-5	6-12
% of IL-17A$^+$ T-cells	Neonates	0.495	0.303	6	2.953	1.899	6	<0.0001				
(Fig. 2H right)	0.5-2 yrs.	0.570	0.395	5	1.136	1.305	5	0.8630	0.0085			
	3-5 yrs.	0.554	0.238	5	1.482	0.174	5	0.5333	0.0492	0.8816		
	6-12 yrs.	0.312	0.166	5	0.848	0.615	5	0.8630	0.0016	0.8816	0.8380	
	Adults	0.282	0.186	5	0.334	0.273	5	0.9207	<0.0001	0.6719	0.2820	0.8630

Rest.-Resting, Neo-Neonates, n-number of donors, p values obtained from Kruskal Wallis test followed by Dunn's post hoc test

**Table S13. Extensive statistical data of T-bet expression in addition to Fig. 2H.**																			
**Study (Corres-ponding figure)**	**Age group**	**Mean**	**SD**	***n***	**Mean**	**SD**	***n***	**Mean**	**SD**	***n***	**Mean**	**SD**	***n***	***P* Value**		***P Value***		***P* Value**	
		**resting**			***S. aureus***			***S. epidermidis***			***B. infantis***			***S. aureus***		***S. epidermidis***		***B. infantis***	
														Rest. vs. *S. aureus*	age vs. *S. aureus*	Rest. vs. *S. epidermidis*	age vs. *S. epidermidis*	Rest. vs. *B.infantis*	age vs. *B. infantis*
% of RORγt$^+$ T-cells	Neonates	0.814	0.496	6	11.02	3.843	6	10.68	4.679	6	3.568	1.925	6	<0.0001		<0.0001		0.0206	
(Fig. 2I)	Adults	0.670	0.641	9	1.902	1.171	9	2.443	0.772	5	2.354	1.150	5	0.0775	<0.0001	0.0165	<0.0001	0.0345	0.6161

Rest.-Resting, Neo-Neonates, n-number of donors, p values obtained from Kruskal Wallis test followed by Dunn's post hoc test

Table S14. Supplementary statistical data to Fig. 3A about the effect of HLA-DR-Blockade on *B. infantis*-induced CD25 expression.

Study (Corresponding figure)	Age group	Mean	SD	*n*	Mean	SD	*n*	*P* Value
		B. infantis			**HLA-DR Blockade**			***B. infantis vs. B. infantis + αHLA-DR***
% of $CD25^+$ T-cells	Neonates	7.524	3.761	5	3.490	2.281	5	0.0493
(Fig. 3A)	0.5-2 yrs.	8.094	3.518	5	3.182	1.812	5	0.0202
	3-5 yrs.	10.51	3.712	5	4.064	1.984	5	0.0029
	6-12 yrs.	8.396	5.409	5	4.046	2.572	5	0.0383
	Adults	11.40	3.876	6	4.000	0.977	6	0.0003

Rest.-Resting, Neo-Neonates, n-number of donors, p values obtained from one-way ANOVA followed by Holm-Sidak post hoc test

Table S15. Genes analysed by RNA-Seq			
**Name**	**ENSEMBLE_ID**	**Gene**	**Description**
FoxP3	ENSG00000049768	AIID, DIETER, IPEX, JM2, PIDX, SCURFIN, XPID	forkhead box P3
IL-10	ENSG00000136634	CSIF, IL-10, IL10A, TGIF	interleukin 10
TGFbeta	ENSG00000105329	TGFB1	transforming growth factor beta 1
Galectin-1	ENSG00000100097	LGALS1	galectin 1
CD25	ENSG00000134460	IL2RA	interleukin 2 receptor subunit alpha
CTLA-4	ENSG00000163599	CTLA4	cytotoxic T-lymphocyte associated protein 4
IL35B	ENSG00000105246	EBI3, IL27B	Epstein-Barr virus induced 3
GITR	ENSMUSG00000041954	Tnfrsf18	tumor necrosis factor receptor superfamily, member 18
LAG-3 (CD223)	ENSG00000089692	LAG3, CD223	lymphocyte activating 3
CD39	ENSG00000138185	ENTPD1, ATPDase, CD39, NTPDase-1, SPG64	ectonucleoside triphosphate diphosphohydrolase 1
IL7R (CD127)	ENSG00000168685	IL7R	interleukin 7 receptor
CD73	ENSG00000135318	NT5E, CALJA, CD73, NT5, eN, eNT	5'-nucleotidase ecto
Helios	ENSG00000030419	IKZF2, Helios, ZNFN1A2	IKAROS family zinc finger 2
STAT5A	ENSG00000126561	MGF, STAT5, STAT5A	signal transducer and activator of transcription 5A
BDCA4	ENSG00000099250	NRP1; CD304, NRP, VEGF165R	neuropilin 1
PD-1	ENSG00000188389	PDCD1, CD279, PD-1, PD1, SLEB2, hSLE1	programmed cell death 1
CD137	ENSG00000125657	TNFSF9, 4-1BB-L, 4-1BBL, CD137L	TNF superfamily member 9
CD103	ENSMUSG00000005947	Itgae, CD103, alpha-E1	integrin subunit alpha E, epithelial-associated

**Table S16. Summarized statistical data of Galectin-1 secretion of activated T-cells in addition to Fig. 3C.**																
**Study (Corres-ponding figure)**	**Age group**	**Mean**	**SD**	***n***	**Mean**	**SD**	***n***	**Mean**	**SD**	***n***	**Mean**	**SD**	***n***	***P* Value**	***P* Value**	
		**resting**			***S. aureus***			***B. infantis***			**αCD3/αCD28**			Rest. vs. *B. infantis*	age vs. *B. infantis*	
															Neo	3-5
Secreted Galectin-1	Neonates	281.3	55.04	6	315.6	64.47	5	455.0	38.79	5	222.6	38.89	5	0.0316		
[pg/ml]	3-5 yrs.	212.9	38.34	8	232.0	9.612	6	373.1	112.0	8	202.3	58.98	6	0.0228	0.0727	
(Fig. 3C)	Adults	241.3	23.05	9	254.6	39.70	9	537.6	242.0	7	253.2	33.89	6	0.0456	0.4381	0.2107

Rest.-Resting, Neo-Neonates, n-number of donors, p values obtained from one-way ANOVA followed by Holm-Sidak post hoc test

**Table S17. Extensive statistical data of IL-10 secretion by *B. infantis* in addition to Fig. 3D.**												
**Study (Corresponding figure)**	**Age group**	**Mean**	**SD**	***n***	**Mean**	**SD**	***n***	***P* Value**	***P* Value**			
		**resting**			***B. infantis***			Rest. vs. *B. infantis*	age vs. *B. infantis*			
									Neo	0.5-2	3-5	6-12
Secreted IL-10 [pg/ml]	Neonates	2.828	1.315	8	61.53	40.45	8	<0.0001				
(Fig. 3D)	0.5-2 yrs.	2.663	0.738	6	18.11	2.887	6	0.1336	0.0042			
	3-5 yrs.	2.218	0.899	6	22.02	3.605	6	0.0615	0.0086	0.9864		
	6-12 yrs.	2.485	0.944	6	16.75	2.673	6	0.1336	0.0034	0.9864	0.9864	
	Adults	2.058	0.893	8	24.47	8.270	8	0.0118	0.0087	0.9849	0.9864	0.9822

Rest.-Resting, Neo-Neonates, n-number of donors, p values obtained from one-way ANOVA followed by Holm-Sidak post hoc test

Table S18. Summarized statistical data of blockade of *B. infantis*-induced proliferation in neonatal T-cells in addition to Fig. 3F (left graph).

Study (Corresponding figure)	Condition	Mean	SD	*n*	***P* Value**		
		**neonatal T-cells**					
					Rest.	*B. infantis*	*B. infantis + αIL-10*
CFSE[lo] T-cell [%]	Resting	0.196	0.255	5			
(Fig. 3F left)	*B. infantis*	8.260	1.469	5	0.0020		
	B. infantis + αIL-10	7.370	1.078	5	0.0035	0.6481	
	B. infantis + αCTLA-4	21.88	5.764	5	<0.0001	<0.0001	<0.0001

Rest.-Resting, n-number of donors, p values obtained from one-way ANOVA followed by Holm-Sidak post hoc test

Table S19. Summarized statistical data of blockade of *B. infantis*-induced proliferation in adult T-cells in addition to Fig. 3F (right graph).

Study (Corresponding figure)	Condition	Mean	SD	*n*	***P* Value**		
		**naïve adult T-cells**					
					rest	*B. infantis*	*B. infantis + αIL-10*
CFSE[lo] T-cell [%]	Resting	0.311	0.205	4			
(Fig. 3F right)	*B. infantis*	6.185	0.764	4	0.0025		
	B. infantis + αIL-10	7.293	2.754	4	0.0010	0.4458	
	B. infantis + αCTLA-4	14.53	2.754	4	0.0003	<0.0001	0.0010

Rest.-Resting, n-number of donors, p values obtained from one-way ANOVA followed by Holm-Sidak post hoc test

**Table S20. Extensive statistical data about the effect of CTLA-4 on *B. infantis*-induced proliferation in addition to Fig. 3G.**										
**Study (Corresponding figure)**	**Condition**	**Mean**	**SD**	***n***	**Mean**	**SD**	***n***	***P* Value**	***P* Value**
		**neonatal T-cells**			**naïve adult T-cells**			**neonatal T-cells**	**naïve adult T-cells**
								Ratio *S. aureus* + CTLA-4/ *S. aureus*	Ratio *S. aureus* + CTLA-4/ *S. aureus*
ratio of proliferating cells	Ratio *B. infantis* + CTLA-4/ *B. infantis*	2.638	0.422	5	2.363	0.474	4	0.0004	0.0189
(Fig.3G)	Ratio *S. aureus* + CTLA-4/ *S. aureus*	1.274	0.136	5	1.343	0.344	4		

Rest.-Resting, n-number of donors, p values obtained from one-way ANOVA followed by Holm-Sidak post hoc test

**Table S21. . Supplementary statistical data of FoxP3 expression in neonatal T-cells in addition to Fig. 3H (left graph).**													
**Study (Corres-ponding figure)**	**Condition**	**Mean**	**SD**	***n***	**Mean**	**SD**	***n***	**Mean**	**SD**	***n***	***P* Value**		
		**resting**			***S. aureus***			***B. infantis***			***Rest. vs. S. aureus***	***Rest. vs B. infantis***	***S. aureus vs B. infantis***
FoxP3+ producers	$FoxP3^+$	0.002	0.002	4	8.374	4.966	4	2.192	1.255	4	0.0006	<0.0001	0.0016
of $CD4^+$ T-cells	$FoxP3^+ROR\gamma t^+$	1.250	2.500	4	0.530	0.746	4	0.583	0.466	4	>0.9999	>0.9999	>0.9999
(Fig. 3H left)	$FoxP3^+$T-bet^+	1.315	2.630	4	1.222	1.092	4	1.052	0.528	4	0.9983	0.9886	>0.9999
	$FoxP3^+$GATA-3^+	0.000	0.000	4	7.854	5.327	4	15.75	1.577	4	0.0003	0.8168	0.0149
	$\Sigma FoxP3^+$ T-cells	2.565	0.741	4	17.98	4.194	4	19.58	7.270	4	0.0026	0.0016	0.4857

Rest.-Resting, Σ-sum, n-number of donors, p values obtained from one-way ANOVA followed by Holm-Sidak post hoc test

**Table S22. Detailed statistical data of FoxP3 expression in naïve adult T-cells in addition to Fig. 3H (right graph).**													
**Study (Corres-ponding figure)**	**Condition**	**Mean**	**SD**	***n***	**Mean**	**SD**	***n***	**Mean**	**SD**	***n***	***P* Value**		
		**resting**			***S. aureus***			***B. infantis***			***Rest. vs. S. aureus***	***Rest. vs B. infantis***	***S. aureus vs B. infantis***
FoxP3$^+$ producers	FoxP3$^+$	0.213	0.426	4	4.559	2.057	4	15.93	1.711	4	<0.0001	<0.0001	<0.0001
of CD4$^+$ T-cells	FoxP3$^+$RORγt$^+$	0.216	0.431	4	0.882	0.463	4	2.734	0.783	4	0.9975	0.0294	0.1457
(Fig. 3H right)	FoxP3$^+$T-bet$^+$	0.000	0.000	4	0.345	0.374	4	0.472	0.374	4	>0.9999	>0.9999	0.9998
	FoxP3$^+$GATA-3$^+$	0.800	1.600	4	3.181	1.507	4	2.377	0.868	4	0.0474	0.4550	0.8239
	ΣFoxP3$^+$ T-cells	1.226	0.344	4	8.967	1.975	4	21.51	7.103	4	0.0004	0.0001	0.0020

Rest.-Resting, Σ-sum, n-number of donors, p values obtained from one-way ANOVA followed by Holm-Sidak post hoc test

Table S23. **Summarized statistical data of proliferation in Cross experiments in neonatal T-cells in addition to Fig. 4B (left graph).**

Study (Corresponding figure)	Condition	Mean	SD	*n*	*P* Value			
		neonatal T-cells						
					Rest.	*S. aureus*	*B. infantis*	*S. aureus + B. infantis*
CFSE[lo] T-cell [%]	Resting	0.2108	0.1035	4				
(Fig. 4B left)	*S. aureus*	24.40	10.02	8	<0.0001			
	B. infantis	5.475	2.999	8	0.5486	<0.0001		
	S. aureus + B. infantis	21.23	5.906	4	0.0006	0.7261	0.0024	
	B. infantis + S. aureus	4.403	2.542	4	0.7261	0.0002	0.7808	0.0043

Rest.-Resting, n-number of donors, p values obtained from one-way ANOVA followed by Holm-Sidak post hoc test

Table S24. **Detailed statistical data of proliferation in Cross experiments in naïve adult T-cells in addition to Fig. 4B (middle graph).**

Study (Corresponding figure)	Condition	Mean	SD	*n*	*P* Value			
		naïve adult T-cells						
					Rest.	*S. aureus*	*B. infantis*	*S. aureus + B. infantis*
CFSE[lo] T-cell [%]	Resting	0.6232	0.1335	5				
(Fig. 4B middle)	*S. aureus*	23.38	9.364	10	<0.0001			
	B. infantis	6.407	1.928	10	0.1881	<0.0001		
	S. aureus + B. infantis	23.26	4.718	7	<0.0001	0.9640	<0.0001	
	B. infantis + S. aureus	9.188	3.744	6	0.0629	0.0001	0.5660	0.0004

Rest.-Resting, n-number of donors, p values obtained from one-way ANOVA followed by Holm-Sidak post hoc test

Table S25. **Supplementary statistical data of proliferation in Cross experiments in memory adult T-cells in addition to Fig. 4B (right graph).**

Study (Corresponding figure)	Condition	Mean	SD	*n*	*P* Value			
		memory adult T-cells						
					Rest.	*S. aureus*	*B. infantis*	*S. aureus + B. infantis*
CFSE[lo] T-cell [%]	Resting	0.6864	0.1293	5				
(Fig. 4B right)	*S. aureus*	33.04	11.09	10	<0.0001			
	B. infantis	9.948	3.589	10	0.0579	<0.0001		
	S. aureus + B. infantis	29.89	6.276	7	<0.0001	0.5797	<0.0001	
	B. infantis + S. aureus	11.24	3.358	6	0.0579	<0.0001	0.7146	0.0001

Rest.-Resting, n-number of donors, p values obtained from one-way ANOVA followed by Holm-Sidak post hoc test

**Table S26.** **Extensive statistical data of CD25 expression in Cross experiments in neonatal T-cells in addition to Fig. 4C (left graph).**								
**Study (Corresponding figure)**	**Condition**	**Mean**	**SD**	***n***	***P* Value**			
		**neonatal T-cells**						
					Rest.	*S. aureus*	*B. infantis*	*S. aureus + B. infantis*
% of CD25⁺ T-cells	Resting	0.2543	0.2876	4				
(Fig. 4C left)	*S. aureus*	29.74	8.567	8	<0.0001			
	B. infantis	6.558	4.768	8	0.2783	<0.0001		
	S. aureus + B. infantis	19.04	9.823	4	0.0058	0.0913	0.0427	
	B. infantis + S. aureus	9.173	6.796	4	0.2248	0.0005	0.5432	0.2041

Rest.-Resting, n-number of donors, p values obtained from one-way ANOVA followed by Holm-Sidak post hoc test

**Table S27.** **Summarized statistical data of CD25 expression in Cross experiments in naïve adult T-cells in addition to Fig. 4C (middle graph).**								
**Study (Corresponding figure)**	**Condition**	**Mean**	**SD**	***n***	***P* Value**			
		**naïve adult T-cells**						
					Rest.	*S. aureus*	*B. infantis*	*S. aureus + B. infantis*
% of CD25⁺ T-cells	Resting	0.9296	1.256	5				
(Fig. 4C middle)	*S. aureus*	31.58	9.315	10	<0.0001			
	B. infantis	7.154	3.410	10	0.2446	<0.0001		
	S. aureus + B. infantis	26.72	7.433	5	<0.0001	0.2719	<0.0001	
	B. infantis + S. aureus	12.43	5.987	5	0.0340	<0.0001	0.2719	0.0075

Rest.-Resting, n-number of donors, p values obtained from one-way ANOVA followed by Holm-Sidak post hoc test

**Table S28.** **Detailed statistical data of CD25 expression in Cross experiments in memory adult T-cells in addition to Fig. 4C (right graph).**								
**Study (Corresponding figure)**	**Condition**	**Mean**	**SD**	***n***	***P* Value**			
		**memory adult T-cells**						
					Rest.	*S. aureus*	*B. infantis*	*S. aureus + B. infantis*
% of CD25⁺ T-cells	Resting	0.351	0.318	5				
(Fig. 4C right)	*S. aureus*	27.52	6.934	10	<0.0001			
	B. infantis	2.344	1.144	10	0.5270	<0.0001		
	S. aureus + B. infantis	24.06	7.484	5	<0.0001	0.4794	<0.0001	
	B. infantis + S. aureus	19.30	8.670	5	<0.0001	0.0496	<0.0001	0.4794

Rest.-Resting, n-number of donors, p values obtained from one-way ANOVA followed by Holm-Sidak post hoc test

Table S29. **Supplementary statistical data of cytokine expression in Cross experiments in neonatal T-cells in addition to Fig. 4D (left graph).**

Study (Corres-ponding figure)	Condition	Mean	SD	*n*	*P* Value			
		neonatal T-cells						
					Rest.	*S. aureus*	*B. infantis*	*S. aureus* + *B. infantis*
Sum of Th1 cytokines [%]	Resting	1.049	1.052	4				
(Fig. 4D left)	*S. aureus*	32.54	12.90	8	0.0002			
	B. infantis	15.72	4.420	8	0.0946	0.0063		
	S. aureus + *B. infantis*	26.54	7.606	4	0.0056	0.5002	0.1955	
	B. infantis + *S. aureus*	14.73	7.470	4	0.1955	0.0194	0.8533	0.1955

Rest.-Resting, n-number of donors, p values obtained from one-way ANOVA followed by Holm-Sidak post hoc test

Table S30. **Summarized statistical data of cytokine expression in Cross experiments in naïve adult T-cells in addition to Fig. 4D (middle graph).**

Study (Corres-ponding figure)	Condition	Mean	SD	*n*	*P* Value			
		naïve adult T-cells						
					Rest.	*S. aureus*	*B. infantis*	*S. aureus* + *B. infantis*
Sum of Th1 cytokines [%]	Resting	0.699	0.558	5				
(Fig. 4D middle)	*S. aureus*	17.13	3.616	10	<0.0001			
	B. infantis	4.559	1.615	10	0.2744	<0.0001		
	S. aureus + *B. infantis*	15.78	8.628	5	<0.0001	0.5506	0.0002	
	B. infantis + *S. aureus*	8.194	3.451	5	0.0342	0.0025	0.2744	0.0325

Rest.-Resting, n-number of donors, p values obtained from one-way ANOVA followed by Holm-Sidak post hoc test

Table S31. **Extensive statistical data of cytokine expression in Cross experiments in memory adult T-cells in addition to Fig. 4D (right graph).**

Study (Corres-ponding figure)	Condition	Mean	SD	*n*	*P* Value			
		memory adult T-cells						
					Rest.	*S. aureus*	*B. infantis*	*S. aureus* + *B. infantis*
Sum of Th1 cytokines [%]	Resting	0.5638	0.4144	5				
(Fig. 4D right)	*S. aureus*	25.21	9.320	10	<0.0001			
	B. infantis	7.514	3.190	10	0.2903	<0.0001		
	S. aureus + *B. infantis*	19.84	5.063	5	0.0006	0.2903	0.0078	
	B. infantis + *S. aureus*	16.61	9.459	5	0.0044	0.0954	0.0582	0.4473

Rest.-Resting, n-number of donors, p values obtained from one-way ANOVA followed by Holm-Sidak post hoc test

**Table S32.** Summarized statistical data of galectin-1 secretion in Cross experiments in neonatal T-cells in addition to Fig. 4E (left graph).								
**Study (Corresponding figure)**	**Condition**	**Mean**	**SD**	***n***	***P* Value**			
		**neonatal T-cells**						
					Rest.	*S. aureus*	*B. infantis*	*S. aureus + B. infantis*
Secreted Galectin-1 [pg/ml]	Resting	270.8	45.02	4				
(Fig. 4E left)	*S. aureus*	300.4	57.46	4	0.7557			
	B. infantis	548.79	60.73	4	0.0001	0.0003		
	S. aureus + B. infantis	375.75	66.28	4	0.1112	0.2802	0.0072	
	B. infantis + S. aureus	522.1	74.21	4	0.0003	0.0009	0.7557	0.0211

Rest.-Resting, n-number of donors, p values obtained from one-way ANOVA followed by Holm-Sidak post hoc test

**Table S33.** Detailed statistical data of galectin-1 secretion in Cross experiments in naïve adult T-cells in addition to Fig. 4D (middle graph).								
**Study (Corresponding figure)**	**Condition**	**Mean**	**SD**	***n***	***P* Value**			
		**naïve adult T-cells**						
					Rest.	*S. aureus*	*B. infantis*	*S. aureus + B. infantis*
Secreted Galectin-1 [pg/ml]	Resting	185.0	28.54	4				
(Fig. 4E middle)	*S. aureus*	248.9	36.67	4	0.3452			
	B. infantis	480.74	105.9	4	0.0004	0.0030		
	S. aureus + B. infantis	298.7	41.27	4	0.1036	0.4601	0.0142	
	B. infantis + S. aureus	546.8	167.2	4	<0.0001	0.0004	0.3298	0.0018

Rest.-Resting, n-number of donors, p values obtained from one-way ANOVA followed by Holm-Sidak post hoc test

**Table S34.** Extensive statistical data of galectin-1 secretion in Cross experiments in memory adult T-cells in addition to Fig. 4E (right graph).								
**Study (Corresponding figure)**	**Condition**	**Mean**	**SD**	***n***	***P* Value**			
		**memory adult T-cells**						
					Rest.	*S. aureus*	*B. infantis*	*S. aureus + B. infantis*
Secreted Galectin-1 [pg/ml]	Resting	189.2	29.12	4				
(Fig. 4E right)	*S. aureus*	265.2	55.46	4	0.1826			
	B. infantis	531.0	124.7	4	<0.0001	0.0002		
	S. aureus + B. infantis	318.52	60.81	4	0.0311	0.3424	0.0014	
	B. infantis + S. aureus	473.2	80.02	4	0.0001	0.0017	0.3043	0.0123

Rest.-Resting, n-number of donors, p values obtained from one-way ANOVA followed by Holm-Sidak post hoc test

**Table S35.** **Summarized statistical data of proliferation in Cross experiments of Corona patients in addition to Fig. 4G (left graph).**								
**Study (Corres-ponding figure)**	**Condition**	**Mean**	**SD**	***n***	***P* Value**			
		**COVID-19 patients**						
					Rest.	Nucleo-capsid pool	*B. infantis*	Nucleo-capsid pool + *B. infantis*
CFSE^{lo} T-cell [%]	Resting	0.773	0.284	5				
(Fig. 4G left)	Nucleocapsid pool	9.752	2.886	5	<0.0001			
	B. infantis	5.932	2.073	5	0.0003	0.0046		
	Nucleocapsid pool + *B. infantis*	5.766	1.951	5	0.0005	0.0033	0.8911	
	B. infantis + Nucleocapsid pool	5.766	1.951	5	0.0021	0.0007	0.4442	0.5283

Rest.-Resting, n-number of donors, p values obtained from one-way ANOVA followed by Holm-Sidak post hoc test

**Table S36.** **Extensive statistical data of IL-10 secretion in Cross experiments of Corona patients in addition to Fig. 4G (middle graph).**								
**Study (Corres-ponding figure)**	**Condition**	**Mean**	**SD**	***n***	***P* Value**			
		**COVID-19 patients**						
					Rest.	Nucleo-capsid pool	*B. infantis*	Nucleocapsid pool + *B. infantis*
no. of IL-10 secreting cells	Resting	9.800	7.190	5				
(Fig. 4G middle)	*Nucleocapsid pool*	8.200	7.791	5	0.9758			
	B. infantis	81.40	34.50	5	0.0101	0.0094		
	Nucleocapsid pool + B. infantis	329.8	125.3	5	<0.0001	<0.0001	0.0010	
	B. infantis + Nucleocapsid pool	139.2	41.10	5	0.0213	0.0199	0.8940	0.0014

Rest.-Resting, n-number of donors, p values obtained from one-way ANOVA followed by Holm-Sidak post hoc test

**Table S37.** **Detailed statistical data of cytokine expression in Cross experiments of Corona patients in addition to Fig. 4G (right graph).**								
**Study (Corres-ponding figure)**	**Condition**	**Mean**	**SD**	***n***	***P* Value**			
		**COVID-19 patients**						
					Rest.	Nucleo-capsid pool	*B. infantis*	Nucleo-capsid pool + *B. infantis*
Th1 cytokine producer [%]	Resting	0.749	0.393	5				
(Fig. 4G right)	*Nucleocapsid pool*	48.59	11.36	5	<0.0001			
	B. infantis	26.69	5.061	5	<0.0001	0.0490		
	Nucleocapsid pool + B. infantis	21.82	3.992	5	<0.0001	0.0038	0.3751	
	B. infantis + Nucleocapsid pool	13.80	5.769	5	0.0085	<0.0001	0.0119	0.0982

Rest.-Resting, n-number of donors, p values obtained from one-way ANOVA followed by Holm-Sidak post hoc test

**Table S38. Supplementary statistical data of Galectin-1 secretion in Cross experiments of Corona patients in addition to Fig. 4H.**								
**Study (Corres-ponding figure)**	**Condition**	**Mean**	**SD**	***n***	***P* Value**			
		**COVID-19 patients**						
					Rest.	Nucleo-capsid pool	*B. infantis*	Nucleo-capsid pool + *B. infantis*
Secreted Galectin-1 [pg/ml]	Resting	202.8	43.13	6				
(Fig. 4H)	*Nucleocapsid pool*	214.1	46.23	6	0.9866			
	B. infantis	513.6	110.8	6	<0.0001	<0.0001		
	Nucleocapsid pool + B. infantis	226.8	47.88	6	0.9778	0.9866	<0.0001	
	B. infantis + Nucleocapsid pool	528.0	121.5	6	<0.0001	<0.0001	0.9866	<0.0001

Rest.-Resting, n-number of donors, p values obtained from one-way ANOVA followed by Holm-Sidak post hoc test

**Table S39. Supplementary statistical of proliferation followed by *living B. infantis* of neonatal T-cells in addition to Fig. 5B (left graph).**								
**Study (Corres-ponding figure)**	**Condition**	**Mean**	**SD**	***n***	***P* Value**			
		**neonatal T-cells**						
					Rest.	*S. aureus*	*B. infantis*	*S. aureus + B. infantis*
CFSE^{lo} T-cell [%]	Resting	1.210	0.805	7				
(Fig. 5B left)	*S. aureus*	85.30	10.22	7	<0.0001			
	B. infantis	6.194	3.776	7	0.5675	<0.0001		
	S. aureus + B. infantis	59.87	31.31	7	<0.0001	0.0061	<0.0001	
	B. infantis + S. aureus	23.17	14.17	7	0.0162	<0.0001	0.0583	0.0002

Rest.-Resting, n-number of donors, p values obtained from one-way ANOVA followed by Holm-Sidak post hoc test

**Table S40. Summarized statistical data of proliferation followed by living B. infantis of naïve adult T-cells in addition to Fig. 5B (right graph).**								
**Study (Corres-ponding figure)**	**Condition**	**Mean**	**SD**	***n***	***P* Value**			
		**naïve adult T-cells**						
					Rest.	*S. aureus*	*B. infantis*	*S. aureus + B. infantis*
CFSE^{lo} T-cell [%]	Resting	1.118	1.261	6				
(Fig. 5B right)	*S. aureus*	43.30	10.54	6	<0.0001			
	B. infantis	4.485	2.510	6	0.5932	<0.0001		
	S. aureus + B. infantis	34.87	20.04	6	<0.0001	0.1874	<0.0001	
	B. infantis + S. aureus	17.42	7.746	6	0.0147	0.0003	0.0481	0.0096

Rest.-Resting, n-number of donors, p values obtained from one-way ANOVA followed by Holm-Sidak post hoc test

Table S41. Detailed statistical data of proliferation in Transwell assays followed by neonatal T-cells in addition to Fig. 5C (left graph).

Study (Corresponding figure)	Condition	Mean	SD	*n*	*P* Value			
		neonatal T-cells						
					Rest.	*S. aureus*	*B. infantis*	*S. aureus + B. infantis*
CFSE^{lo} T-cell [%]	Resting	1.528	1.584	5				
(Fig. 5C left)	*S. aureus*	75.36	13.94	5	<0.0001			
	B. infantis	4.708	1.926	5	0.8392	<0.0001		
	S. aureus + B. infantis	96.36	13.48	5	<0.0001	0.7595	<0.0001	
	B. infantis + S. aureus	5.810	3.825	5	0.8349	<0.0001	0.8469	<0.0001

Rest.-Resting, n-number of donors, p values obtained from one-way ANOVA followed by Holm-Sidak post hoc test

Table S42. Extensive statistical data of proliferation in Transwell assays followed by naïve adult T-cells in addition to Fig. 5C (right graph).

Study (Corresponding figure)	Condition	Mean	SD	*n*	*P* Value			
		naïve adult T-cells						
					Rest.	*S. aureus*	*B. infantis*	*S. aureus + B. infantis*
CFSE^{lo} T-cell [%]	Resting	0.557	0.410	6				
(Fig. 5C right)	*S. aureus*	48.40	15.95	6	<0.0001			
	B. infantis	3.858	2.698	6	0.9420	<0.0001		
	S. aureus + B. infantis	49.22	16.53	6	<0.0001	0.9886	<0.0001	
	B. infantis + S. aureus	4.595	3.195	6	0.9420	<0.0001	0.9886	<0.0001

Rest.-Resting, n-number of donors, p values obtained from one-way ANOVA followed by Holm-Sidak post hoc test

Table S43. Summarized data of CD16 expression by monocytes (Fig. S1A, upper left).																				
**Study (Corresponding figure)**	**Condition**	**Mean**	**SD**	***n***	**Mean**	**SD**	***n***	**Mean**	**SD**	***n***	***P* Value**			***P* Value**			***P* Value**			
		**neonatal T-cells**			**naïve T-cells of children**			**naïve adult T-cells**			**neonatal T-cells**			**naïve T-cells of children**			**naïve adult T-cells**			
											Rest.	*S. aureus*	*S. epidermidis*	Rest.	*S. aureus*	*S. epidermidis*	Rest.	*S. aureus*	*S. epidermidis*	
CD16 expression	Resting	0,238	0,162	7	0,477	0,175	5	0,439	0,305	8										
(Fig. S1A, upper left)	*S. aureus*	45,97	14,07	7	45,56	17,80	5	40,34	16,34	8	0,0014			0,0192			0,0002			
	S. epidermidis	38,86	15,62	7	44,84	7,57	5	28,96	12,56	8	0,0168	>0,9999		0,0264	>0,9999		0,0137	>0,9999		
	B. infantis	38,33	12,51	7	44,12	11,36	5	28,74	10,29	8	0,0230	>0,9999	>0,9999	0,0226	>0,9999	>0,9999	0,0156	>0,9999	>0,9999	

Rest.-Resting, n-number of donors, p values obtained from Kruskal-Wallis test followed by Dunn's post hoc test

Table S44. Summarized data of HLA-DR expression by monocytes (Fig. S1A, upper right).																			
Study (Corres-ponding figure)	Condition	Mean	SD	*n*	Mean	SD	*n*	Mean	SD	*n*	*P* Value			*P* Value			*P* Value		
		neonatal T-cells			naïve T-cells of children			naïve adult T-cells			neonatal T-cells			naïve T-cells of children			naïve adult T-cells		
											Rest.	*S. aureus*	*S. epidermidis*	Rest.	*S. aureus*	*S. epidermidis*	Rest.	*S. aureus*	*S. epidermidis*
HLA-DR expression	Resting	0,404	0,313	7	0,245	0,098	5	0,355	0,236	8									
(Fig. S1A, upper right)	*S. aureus*	3,911	1,169	7	4,831	2,070	5	3,134	1,789	8	<0,0001			0,0161			0,0063		
	S. epidermidis	3,294	1,416	7	4,304	2,340	5	3,341	2,172	8	0,0004	0,7010		0,0314	0,9225		0,0038	0,7921	
	B. infantis	3,469	1,399	7	4,088	2,641	5	2,042	1,288	8	0,0003	0,7334	0,7819	0,0356	0,9225	0,9225	0,1445	0,3115	0,2839

Rest.-Resting, n-number of donors, p values obtained from one-way ANOVA test followed by Holm-Sidak post hoc test

Table S45. Summarized data of IL-1ß expression by monocytes (Fig. S1A, lower left).																				
**Study (Corres-ponding figure)**	**Condition**	**Mean**	**SD**	***n***	**Mean**	**SD**	***n***	**Mean**	**SD**	***n***	***P* Value**			***P* Value**			***P* Value**		
		**neonatal T-cells**			**naïve T-cells of children**			**naïve adult T-cells**			**neonatal T-cells**			**naïve T-cells of children**			**naïve adult T-cells**		
											Rest.	*S. aureus*	*S. epidermidis*	Rest.	*S. aureus*	*S. epidermidis*	Rest.	*S. aureus*	*S. epidermidis*
IL-1ß expression	Resting	0,357	0,104	7	0,275	0,104	5	0,331	0,223	7									
(Fig. S1A, lower left)	*S. aureus*	3,144	1,237	7	3,050	0,825	5	2,894	1,029	7	0,0040			0,0023			0,0053		
	S. epidermidis	2,877	1,575	7	3,226	1,120	5	2,643	1,588	7	0,0080	0,7165		0,0016	0,9486		0,0112	0,8316	
	B. infantis	3,873	1,837	7	2,865	1,433	5	2,273	1,662	7	0,0004	0,5457	0,4553	0,0034	0,9486	0,9239	0,0330	0,7473	0,8316

Rest.-Resting, n-number of donors, p values obtained from one-way ANOVA test followed by Holm-Sidak post hoc test

Table S46. Summarized data of IL-6 expression by monocytes (Fig. S1A, lower right).

Study (Corresponding figure)	Condition	Mean	SD	*n*	Mean	SD	*n*	Mean	SD	*n*	*P* Value			*P* Value			*P* Value		
		neonatal T-cells			naïve T-cells of children			naïve adult T-cells			neonatal T-cells			naïve T-cells of children			naïve adult T-cells		
											Rest.	*S. aureus*	*S. epidermidis*	Rest.	*S. aureus*	*S. epidermidis*	Rest.	*S. aureus*	*S. epidermidis*
IL-6 expression	Resting	0,552	0,160	7	0,416	0,161	5	0,492	0,216	7									
(Fig. S1A, lower right)	*S. aureus*	3,353	1,483	7	3,366	1,668	5	3,060	1,203	7	0,0018			0,0489			0,0062		
	S. epidermidis	2,687	1,344	7	3,549	1,248	5	2,965	1,904	7	0,0150	>0,9999		0,0166	>0,9999		0,0230	>0,9999	
	B. infantis	2,739	1,382	7	3,092	1,383	5	3,575	1,969	7	0,0206	>0,9999	>0,9999	0,0969	>0,9999	>0,9999	0,0044	>0,9999	>0,9999

Rest.-Resting, n-number of donors, p values obtained from Kruskal-Wallis test followed by Dunn's post hoc test

Table S47. Summarized data of IL-6 secretion by monocytes (Fig. S1B).

Study (Corres-ponding figure)	Condition	Mean	SD	*n*	Mean	SD	*n*	Mean	SD	*n*	*P* Value			*P* Value			*P* Value		
		neonatal T-cells			naïve T-cells of children			naïve adult T-cells			neonatal T-cells			naïve T-cells of children			naïve adult T-cells		
											Rest.	*S. aureus*	*S. epidermidis*	Rest.	*S. aureus*	*S. epidermidis*	Rest.	*S. aureus*	*S. epidermidis*
IL-6 secretion	Resting	125,90	93,35	14	98,75	62,29	16	5,98	5,12	16									
(Fig. S1B)	*S. aureus*	2670,00	279,30	14	2735,00	662,10	16	2707,00	279,80	16	<0,0001			<0,0001			<0,0001		
	S. epidermidis	2669,00	322,60	14	2556,00	324,40	16	2735,00	265,40	16	<0,0001	>0,9999		<0,0001	>0,9999		<0,0001	>0,9999	
	B. infantis	2484,00	550,40	14	2566,00	319,40	10	2728,00	574,60	16	<0,0001	>0,9999	>0,9999	0,0004	>0,9999	>0,9999	<0,0001	>0,9999	>0,9999

Rest.-Resting, n-number of donors, p values obtained from Kruskal-Wallis test followed by Dunn's post hoc test

Table S48. Summarised data on the survival of CD4+ T-cells (Fig. S2C).

**Study (Corres-ponding figure)**	**Condition**	**Mean**	**SD**	***n***	**Mean**	**SD**	***n***	**Mean**	**SD**	***n***	***P* Value**		***P* Value**		***P* Value**	
		**neonatal T-cells**			**naïve T-cells of children**			**naïve adult T-cells**			**neonatal T-cells**		**naïve T-cells of children**		**naïve adult T-cells**	
											S. aureus	*S. epidermidis*	*S. aureus*	*S. epidermidis*	*S. aureus*	*S. epidermidis*
Live cell frequency	*S. aureus*	40,90	8,90	6	38,92	22,80	5	36,70	9,24	6						
(Fig. S2C)	*S. epidermidis*	41,28	14,68	6	38,00	16,58	5	37,87	6,59	6	0,9611		0,9600		0,9979	
	B. infantis	40,20	4,53	3	40,17	3,41	3	41,15	10,83	4	0,9456	0,9611	0,9600	0,9868	0,9979	0,9979

Rest.-Resting, n-number of donors, p values obtained from one-way ANOVA test followed by Holm-Sidak post hoc test

Table S49. Supplementary statistical data to Fig. S2E about the influence of HLA-DR inhibition on T-cell activation.

Study (Corresponding figure)	Condition	Mean	SD	*n*	Mean	SD	*n*	Mean	SD	*n*	*P* Value		*P* Value	
**neonatal T-cells**														
		resting			*S. aureus*			*S. epidermidis*			*S. aureus*		*B. infantis*	
											Rest. vs. *S. aureus*	*S. aureus* Ø αHLA-DR vs. *S. aureus* + αHLA-DR	Rest. vs. *S. epidermidis*	*S. epidermidis* Ø αHLA-DR vs. *S. epidermidis* + αHLA-DR
% of $CD25^+$ T-cells	Ø αHLA-DR	0,8308	0,4792	5	24,56	10,04	5	5,020	1,576	5	<0,0001		0,3814	
(Fig.S2E)	+ αHLA-DR	0,6694	0,3926	5	6,002	2,963	5	1,730	0,711	5	0,2109	<0,0001	0,8994	0,507
**naive T cells from infants aged between 0.5 -2 years**														
Study (Corresponding figure)	Condition	Mean	SD	*n*	Mean	SD	*n*	Mean	SD	*n*	*P* Value		*P* Value	
		resting			*S. aureus*			*S. epidermidis*			*S. aureus*		*B. infantis*	
											Rest. vs. *S. aureus*	*S. aureus* Ø αHLA-DR vs. *S. aureus* + αHLA-DR	Rest. vs. *S. epidermidis*	*S. epidermidis* Ø αHLA-DR vs. *S. epidermidis* + αHLA-DR
% of $CD25^+$ T-cells	Ø αHLA-DR	1,231	0,868	4	24,40	7,906	4	3,873	0,516	4	<0,0001		0,6031	
(Fig.S2E)	+ αHLA-DR	1,105	0,699	4	4,123	0,886	4	1,145	0,572	4	0,5577	<0,0001	0,9973	0,6031
**naive T cells from children aged between 3-5 years**														
Study (Corresponding figure)	Condition	Mean	SD	*n*	Mean	SD	*n*	Mean	SD	*n*	*P* Value		*P* Value	
		resting			*S. aureus*			*S. epidermidis*			*S. aureus*		*B. infantis*	
											Rest. vs. *S. aureus*	*S. aureus* Ø αHLA-DR vs. *S. aureus* + αHLA-DR	Rest. vs. *S. epidermidis*	*S. epidermidis* Ø αHLA-DR vs. *S. epidermidis* + αHLA-DR
% of $CD25^+$ T-cells	Ø αHLA-DR	1,377	0,4918	4	28,18	12,18	4	13,45	5,026	4	<0,0001		0,0038	
(Fig.S2E)	+ αHLA-DR	1,347	0,5667	4	7,948	3,614	4	5,288	0,871	4	0,1381	<0,0001	0,4039	0,0437
**naive T cells from children aged between 6-12 years**														
Study (Corresponding figure)	Condition	Mean	SD	*n*	Mean	SD	*n*	Mean	SD	*n*	*P* Value		*P* Value	
		resting			*S. aureus*			*S. epidermidis*			*S. aureus*		*B. infantis*	
											Rest. vs. *S. aureus*	*S. aureus* Ø αHLA-DR vs. *S. aureus* + αHLA-DR	Rest. vs. *S. epidermidis*	*S. epidermidis* Ø αHLA-DR vs. *S. epidermidis* + αHLA-DR
% of $CD25^+$ T-cells	Ø αHLA-DR	1,737	1,331	4	24,10	12,49	4	15,15	6,292	4	<0,0001		0,0030	
(Fig.S2E)	+ αHLA-DR	1,781	1,627	4	6,258	1,617	4	4,853	3,047	4	0,3877	0,0003	0,3941	0,0304
**naive adult T cells**														
Study (Corresponding figure)	Condition	Mean	SD	*n*	Mean	SD	*n*	Mean	SD	*n*	*P* Value		*P* Value	
		resting			*S. aureus*			*S. epidermidis*			*S. aureus*		*B. infantis*	
											Rest. vs. *S. aureus*	*S. aureus* Ø αHLA-DR vs. *S. aureus* + αHLA-DR	Rest. vs. *S. epidermidis*	*S. epidermidis* Ø αHLA-DR vs. *S. epidermidis* + αHLA-DR
% of $CD25^+$ T-cells	Ø αHLA-DR	0,898	0,779	6	26,78	11,13	6	20,25	7,383	6	<0,0001		<0,0001	
(Fig.S2E)	+ αHLA-DR	1,040	0,796	6	5,620	3,450	6	4,890	2,392	6	0,2469	<0,0001	0,2469	<0,0001

Rest.-Resting, n-number of donors, p values obtained from one-way ANOVA test followed by Holm-Sidak post hoc test

Table S50. Summary of statistical data on the proliferation of T cells in the different age groups (Fig. S2G).

Study (Corres-ponding figure)	Condition	Mean	SD	*n*	Mean	SD	*n*	Mean	SD	*n*	Mean	SD	*n*	Mean	SD	*n*
		neonatal T-cells			naïve T cells from infants aged between 0.5 -2 years			naïve T cells from children aged between 3-5 years			naïve T cells from children aged between 6-12 years			naïve adult T-cells		
IL-6 secretion	Resting	0,5759	0,3046	7	1,055	0,6147	8	1,312	0,589	5	1,155	0,615	6	1,240	1,062	9
(Fig. S1B)	*S. aureus*	46,3	8,737	6	44,8	5,214	3	20,53	3,722	3	24,35	6,628	4	16,12	7,080	9
	S. epidermidis	42,15	6,409	4	16,73	5,369	3	12,53	6,563	3	21,45	4,723	4	11,95	3,079	4
	B. infantis	7,979	3,351	7	4,426	3,053	8	4,146	2,705	5	3,315	3,278	6	5,160	2,875	6

Study (Corres-ponding figure)	Condition	***P* Value**			***P* Value**			***P* Value**			***P* Value**			***P* Value**		
		neonatal T-cells			naïve T cells from infants aged between 0.5 -2 years			naïve T cells from children aged between 3-5 years			naïve T cells from children aged between 6-12 years			naïve adult T-cells		
		Rest.	*S. aureus*	*S. epidermidis*	Rest.	*S. aureus*	*S. epidermidis*	Rest.	*S. aureus*	*S. epidermidis*	Rest.	*S. aureus*	*S. epidermidis*	Rest.	*S. aureus*	*S. epidermidis*
IL-6 secretion	Resting															
(Fig. S1B)	*S. aureus*	<0,0001			<0,0001			<0,0001			<0,0001			<0,0001		
	S. epidermidis	<0,0001	0,2437		<0,0001	<0,0001		0,0033	0,0304		<0,0001	0,5362		0,0022	0,2066	
	B. infantis	0,0349	<0,0001	<0,0001	0,047	<0,0001	<0,0001	0,2209	0,0002	0,0187	0,5362	<0,0001	<0,0001	0,2066	0,0005	0,0792

Rest.-Resting, n-number of donors, p values obtained from one-way ANOVA test followed by Holm-Sidak post hoc test

Table S51. Supplementary statistical data to Fig. S3A about the influence of MyD88 inhibition on CD25 expression.													
Study (Corres-ponding figure)	Condition	Mean	SD	*n*	Mean	SD	*n*	Mean	SD	*n*	***P*** **Value**		***P*** **Value**	
		**resting**			***S. aureus***			***B. infantis***			***S. aureus***		***B. infantis***	
											Rest. vs. *S. aureus*	*S. aureus* Ø MyD88-Inhibitor vs. *S. aureus* + MyD88-Inhibitor	Rest. vs. *B.infantis*	*B. infantis* Ø MyD88-Inhibitor vs. *B. infantis* + MyD88-Inhibitor
% of CD25^{+} T-cells	Ø MyD88-Inhibitor	0,742	0,147	5	29,34	8,431	5	8,413	3,060	5	<0,0001		0,0921	
(Fig. S3A)	+ MyD88-Inhibitor	0,529	0,160	5	24,72	8,492	5	8,391	2,592	5	<0,0001	0,3097	0,0921	0,9948

Rest.-Resting, n-number of donors, p values obtained from one-way ANOVA test followed by Holm-Sidak post hoc test

Table S52. Supplementary statistical data to Fig. S3A about the influence of MyD88 inhibition on IL-2 expression.

Study (Corresponding figure)	Condition	Mean	SD	*n*	Mean	SD	*n*	Mean	SD	*n*	***P* Value**		***P* Value**	
		resting			***S. aureus***			***B. infantis***			***S. aureus***		***B. infantis***	
											Rest. vs. *S. aureus*	*S. aureus* Ø MyD88-Inhibitor vs. *S. aureus* + MyD88-Inhibitor	Rest. vs. *B.infantis*	*B. infantis* Ø MyD88-Inhibitor vs. *B. infantis* + MyD88-Inhibitor
% of IL-2^+ T-cells	Ø MyD88-Inhibitor	0,399	0,137	5	4,95	0,817	5	1,954	0,739	5	<0,0001		0,0056	
(Fig. S3A)	+ MyD88-Inhibitor	0,402	0,230	5	4,68	0,840	5	2,034	0,646	5	<0,0001	0,8002	0,0049	0,8557

Rest.-Resting, n-number of donors, p values obtained from one-way ANOVA test followed by Holm-Sidak post hoc test

Table S53. Supplementary statistical data to Fig. S3A about the influence of MyD88 inhibition on IFNγ expression.

Study (Corresponding figure)	Condition	Mean	SD	*n*	Mean	SD	*n*	Mean	SD	*n*	***P* Value**		***P* Value**	
		resting			***S. aureus***			***B. infantis***			***S. aureus***		***B. infantis***	
											Rest. vs. *S. aureus*	*S. aureus* Ø MyD88-Inhibitor vs. *S. aureus* + MyD88-Inhibitor	Rest. vs. *B.infantis*	*B. infantis* Ø MyD88-Inhibitor vs. *B. infantis* + MyD88-Inhibitor
% of IFNγ⁺ T-cells	Ø MyD88-Inhibitor	0,212	0,052	5	6,62	1,908	5	2,146	0,504	5	<0,0001		0,1253	
(Fig. S3A)	+ MyD88-Inhibitor	0,205	0,162	5	6,45	2,185	5	2,136	0,478	5	<0,0001	0,9746	0,1253	0,9907

Rest.-Resting, n-number of donors, p values obtained from one-way ANOVA test followed by Holm-Sidak post hoc test

Table S54. Supplementary statistical data to Fig. S3B about the influence of αh-CR3α inhibition on T-cell activation.									
**neonatal T-cells**									
**Study (Corres-ponding figure)**	**Condition**	**Mean**	**SD**	***n***	**Mean**	**SD**	***n***	***P* Value**	
		**resting**			***S. aureus***			***S. aureus***	
								Rest. vs. *S. aureus*	*S. aureus Ø αh-CR3α vs. S. aureus + αh-CR3α*
% of CD25$^+$ T-cells	Ø αh-CR3α	0,717	0,335	4	30,33	6,306	4	<0,0001	
(Fig.S2E)	+ αh-CR3α	0,745	0,540	4	25,98	3,170	4	<0,0001	0,0979
**naive T cells from infants aged between 0.5 -2 years**									
**Study (Corres-ponding figure)**	**Condition**	**Mean**	**SD**	***n***	**Mean**	**SD**	***n***	***P* Value**	
		**resting**			***S. aureus***			***S. aureus***	
								Rest. vs. *S. aureus*	*S. aureus Ø αh-CR3α vs. S. aureus + αh-CR3α*
% of CD25$^+$ T-cells	Ø αh-CR3α	1,139	0,655	4	24,03	9,482	4	<0,0001	
(Fig.S2E)	+ αh-CR3α	0,575	0,421	4	23,30	9,695	4	<0,0001	0,9816
**naive adult T cells**									
**Study (Corres-ponding figure)**	**Condition**	**Mean**	**SD**	***n***	**Mean**	**SD**	***n***	***P* Value**	
		**resting**			***S. aureus***			***S. aureus***	
								Rest. vs. *S. aureus*	*S. aureus Ø αh-CR3α vs. S. aureus + αh-CR3α*
% of CD25$^+$ T-cells	Ø αh-CR3α	0,385	0,505	3	22,40	4,40	3	0,0004	
(Fig.S2E)	+ αh-CR3α	1,213	0,416	3	22,07	8,442	3	0,0006	0,9816

Rest.-Resting, n-number of donors, p values obtained from one-way ANOVA test followed by Holm-Sidak post hoc test

Table S55. Summarized statistical data of multiple cytokine producers by *S. epidermidis* in addition to Fig. S4 (upper row).																		
**Study (Corres-ponding figure)**	**Age group**	**Mean**	**SD**	***n***	**Mean**	**SD**	***n***	**Mean**	**SD**	***n***	**Mean**	**SD**	***n***	***P* Value**				
		Single producers			***Double producers***			***Triple producers***			***Non producers***			**age vs. *S. aureus***				
														Neonates	0.5-2 yrs.	3-5 yrs.	6-12 yrs.	
% of cytokine	Neonates	8,223	3,528	4	2,511	3,372	4	0,000	0,000	4	89,27	6,895	4					
producing T-cells	0.5-2 yrs.	6,081	5,577	6	1,217	1,831	6	0,209	0,348	6	92,49	7,735	6	0,368				
	3-5 yrs.	7,678	9,565	6	1,987	3,949	6	0,069	0,116	6	90,27	13,57	6	1,000	0,621			
	6-12 yrs.	8,952	8,170	6	1,431	2,698	6	0,012	0,015	6	89,61	10,75	6	0,621	1,000	1,000		
	Adults	5,223	3,460	5	2,993	3,166	5	0,243	0,541	5	91,54	6,740	5	1,000	0,621	1	0,621	

Rest.-Resting, Neo-Neonates, n-number of donors, p values obtained from Fisher's Exact Test

Table S56. Summarized statistical data of multiple cytokine producers by *B. infantis* in addition to Fig. S4 (lower row).																	
**Study (Corres-ponding figure)**	**Age group**	**Mean**	**SD**	***n***	**Mean**	**SD**	***n***	**Mean**	**SD**	***n***	**Mean**	**SD**	***n***	***P* Value**			
		Single producers			***Double producers***			***Triple producers***			***Non producers***			**age vs. *S. aureus***			
														Neonates	0.5-2 yrs.	3-5 yrs.	6-12 yrs.
% of cytokine	Neonates	2,974	0,355	4	0,211	0,234	4	0,019	0,032	4	96,80	0,431	4				
producing T-cells	0.5-2 yrs.	3,805	1,367	4	0,304	0,088	4	0,060	0,024	4	95,83	1,378	4	1,000			
	3-5 yrs.	3,126	1,307	4	0,267	0,226	4	0,037	0,041	4	96,57	1,530	4	1,000	1,000		
	6-12 yrs.	2,876	1,051	4	0,199	0,133	4	0,011	0,023	4	96,91	1,173	4	1,000	1,000	1,000	
	Adults	1,644	0,506	5	0,203	0,230	5	0,000	0,000	5	98,15	0,411	5	1,000	1,000	1,000	1,000

Rest.-Resting, Neo-Neonates, n-number of donors, p values obtained from Fisher's Exact Test

Table S57. Summarised data on the survival of CD4+ T-cells (Fig. S7A).											
**Study (Corres-ponding figure)**	**Condition**	**Mean**	**SD**	***n***	**Mean**	**SD**	***n***	***P* Value**		***P* Value**	
		**neonatal T-cells**			**naïve adult T-cells**			**neonatal T-cells**		**naïve adult T-cells**	
								resting	*B. infantis*	resting	*B. infantis*
IL-10 secretion	resting	0,912	0,264	6	0,915	0,459	4				
(Fig. S2C)	*B. infantis*	84,22	35,12	6	113,40	41,34	4	<0,0001		<0,0001	
	B. infantis + αIL-10	2,963	1,973	6	1,283	0,790	4	0,9983	<0,0001	>0,9999	<0,0001

Rest.-Resting, n-number of donors, p values obtained from one-way ANOVA test followed by Holm-Sidak post hoc test

**Table S58. Summarized statistical data of blockade of *B. infantis*- induced CD25 expression in neonatal T-cells in addition to Fig. S7B (upper left graph).**							
**Study (Corresponding figure)**	**Condition**	**Mean**	**SD**	***n***	***P* Value**		
		**neonatal T-cells**					
					Rest.	*B. infantis*	*B. infantis + αIL-10*
% of CD25$^+$ T-cells	Resting	0,676	0,414	6			
	B. infantis	6,921	1,727	6	0,0007		
	B. infantis + αIL-10	6,878	2,269	6	0,0007	0,9827	
	B. infantis + αCTLA-4	9,996	2,582	6	<0,0001	0,1414	0,1737

Rest.-Resting, n-number of donors, p values obtained from one-way ANOVA followed by Holm-Sidak post hoc test

**Table S59. Summarized statistical data of blockade of *B. infantis*- induced CD25 expression in adult T-cells in addition to Fig. S7B (upper right graph).**							
**Study (Corres-ponding figure)**	**Condition**	**Mean**	**SD**	***n***	***P* Value**		
		**naïve adult T-cells**					
					rest	*B. infantis*	*B. infantis + αIL-10*
% of $CD25^+$ T-cells	Resting	0,567	0,337	4			
	B. infantis	10,95	4,111	4	0,0049		
	B. infantis + αIL-10	11,35	0,858	4	0,0043	0,9621	
	B. infantis + αCTLA-4	20,07	5,811	4	<0,0001	0,0108	0,0111

Rest.-Resting, n-number of donors, p values obtained from one-way ANOVA followed by Holm-Sidak post hoc test

**Table S60. Summarized statistical data of blockade of *B. infantis*- induced Th1 cytokine expression in neonatal T-cells in addition to Fig. S7B (lower left graph).**							
**Study (Corres-ponding figure)**	**Condition**	**Mean**	**SD**	***n***	***P* Value**		
		**neonatal T-cells**					
					Rest.	*B. infantis*	*B. infantis + αIL-10*
% of $Th1^+$ T-cells	Resting	1,270	0,399	6			
	B. infantis	11,20	1,666	6	<0,0001		
	B. infantis + αIL-10	11,52	1,604	6	<0,0001	0,7706	
	B. infantis + αCTLA-4	15,06	3,100	6	<0,0001	0,0042	0,0058

Rest.-Resting, n-number of donors, p values obtained from one-way ANOVA followed by Holm-Sidak post hoc test

**Table S61. Summarized statistical data of blockade of *B. infantis*- induced Th1 cytokine expression in adult T-cells in addition to Fig. S7B (lower right graph).**							
**Study (Corres-ponding figure)**	**Condition**	**Mean**	**SD**	***n***	***P* Value**		
		**naïve adult T-cells**					
					rest	*B. infantis*	*B. infantis + αIL-10*
% of $Th1^{+}$ T-cells	Resting	0,915	0,190	4			
	B. infantis	12,55	1,532	4	0,0003		
	B. infantis + αIL-10	10,88	1,954	4	0,0011	0,4396	
	B. infantis + αCTLA-4	18,65	5,720	4	<0,0001	0,0219	0,0065

Rest.-Resting, n-number of donors, p values obtained from one-way ANOVA followed by Holm-Sidak post hoc test

Table S62. Summarized statistical data of blockade of *B. infantis*- induced IL-17A expression in neonatal T-cells in addition to Fig. S7C (left graph).

**Study (Corres-ponding figure)**	**Condition**	**Mean**	**SD**	***n***	***P* Value**	
		**neonatal T-cells**				
					Rest.	*B. infantis*
% of IL-17A^{+} T-cells	Resting	0,444	0,140	6		
	B. infantis	8,207	3,024	6	<0,0001	
	B. infantis + αIL-10	8,418	2,951	6	<0,0001	0,6322

Rest.-Resting, n-number of donors, p values obtained from one-way ANOVA followed by Holm-Sidak post hoc test

**Table S63. Summarized statistical data of blockade of *B. infantis*- induced IL-17A expression in neonatal T-cells in addition to Fig. S7C (right graph).**						
**Study (Corres-ponding figure)**	**Condition**	**Mean**	**SD**	***n***	***P* Value**	
		**naive adult T-cells**				
					Rest.	*B. infantis*
% of IL-17A^{+} T-cells	Resting	0,379	0,148	4		
	B. infantis	2,092	1,492	4	0,4671	
	B. infantis + αIL-10	2,209	1,751	4	0,4671	0,9238

Rest.-Resting, n-number of donors, p values obtained from one-way ANOVA followed by Holm-Sidak post hoc test

Table S64. Summarised data on Galectin secretion of CD4+ T-cells (Fig. S7D).											
**Study (Corres-ponding figure)**	**Condition**	**Mean**	**SD**	***n***	**Mean**	**SD**	***n***	***P* Value**		***P* Value**	
		**neonatal T-cells**			**naïve adult T-cells**			**neonatal T-cells**		**naïve adult T-cells**	
								resting	*B. infantis*	resting	*B. infantis*
Galectin-1 secretion	resting	166,20	16,87	6	225,70	34,67	4				
(Fig. S2C)	*B. infantis*	443,00	82,61	6	576,30	113,60	4	<0,0001		<0,0001	
	B. infantis + αICTLA-4	360,00	48,78	6	481,50	41,31	4	<0,0001	0,0479	<0,0001	0,0479

Rest.-Resting, n-number of donors, p values obtained from one-way ANOVA test followed by Holm-Sidak post hoc test
