# Supplementary material for: Bifidobacteria shape antimicrobial T-helper cell responses during infancy and adulthood
Source: Nat Commun. 2023 Sep 23;14:5943. doi: 10.1038/s41467-023-41630-x (PMC10517955; doi:10.1038/s41467-023-41630-x)
Supplement: Supplementary file 3 — Reporting Summary [file 41467_2023_41630_MOESM3_ESM.pdf]

nature portfolio

Corresponding author(s): Brunner-Weinzierl

Last updated by author(s): Aug 9, 2023

Reporting Summary

Nature Portfolio wishes to improve the reproducibility of the work that we publish. This form provides structure for consistency and transparency in reporting. For further information on Nature Portfolio policies, see our Editorial Policies and the Editorial Policy Checklist.

Statistics

For all statistical analyses, confirm that the following items are present in the figure legend, table legend, main text, or Methods section.

n/a	Confirmed	
☐	☒	The exact sample size (n) for each experimental group/condition, given as a discrete number and unit of measurement
☐	☒	A statement on whether measurements were taken from distinct samples or whether the same sample was measured repeatedly
☐	☒	The statistical test(s) used AND whether they are one- or two-sided<br>*Only common tests should be described solely by name; describe more complex techniques in the Methods section.*
☐	☒	A description of all covariates tested
☐	☒	A description of any assumptions or corrections, such as tests of normality and adjustment for multiple comparisons
☐	☒	A full description of the statistical parameters including central tendency (e.g. means) or other basic estimates (e.g. regression coefficient) AND variation (e.g. standard deviation) or associated estimates of uncertainty (e.g. confidence intervals)
☐	☒	For null hypothesis testing, the test statistic (e.g. F, t, r) with confidence intervals, effect sizes, degrees of freedom and P value noted<br>*Give P values as exact values whenever suitable.*
☒	☐	For Bayesian analysis, information on the choice of priors and Markov chain Monte Carlo settings
☒	☐	For hierarchical and complex designs, identification of the appropriate level for tests and full reporting of outcomes
☐	☒	Estimates of effect sizes (e.g. Cohen's d, Pearson's r), indicating how they were calculated

Our web collection on statistics for biologists contains articles on many of the points above.

Software and code

Policy information about availability of computer code

Data collection	FlowJo (version 10.8.1), LEGENDplex Data Analysis Software Suite, FACS Diva software (version 9.0.1), FASTQC tool, Trim Galore! wrapper tool, short read aligner STAR, R package “Rsubread”, R package “bioMaRt", R package “edgeR”, R package “clusterProfiler”, GraphPad Prism 9, Jamovi (version 2.2.21)
Data analysis	No unpublished custom computer code or algorithm was used.

For manuscripts utilizing custom algorithms or software that are central to the research but not yet described in published literature, software must be made available to editors and reviewers. We strongly encourage code deposition in a community repository (e.g. GitHub). See the Nature Portfolio guidelines for submitting code & software for further information.

Data

Policy information about availability of data

All manuscripts must include a data availability statement. This statement should provide the following information, where applicable:

- Accession codes, unique identifiers, or web links for publicly available datasets
- A description of any restrictions on data availability
- For clinical datasets or third party data, please ensure that the statement adheres to our policy

The mRNA sequencing data that has been generated in this study is deposited in the Gene Expression Omnibus database entitled “mRNAseq profiling of

Bifidobacterium longum-stimulated neonatal and adult T-cells” under accession code GSE210336 [https://www.ncbi.nlm.nih.gov/geo/query/acc.cgi?acc=GSE210336].

Research involving human participants, their data, or biological material

Policy information about studies with human participants or human data. See also policy information about sex, gender (identity/presentation), and sexual orientation and race, ethnicity and racism.

Reporting on sex and gender	Information on the sex of the human participants can be found in tables S1-S3. Furthermore, no analysis were performed based on sex. Gender information was not collected.
Reporting on race, ethnicity, or other socially relevant groupings	The grouping of the human participants was based exclusively on age. No information on race or ethnicity was collected.
Population characteristics	For a description of the study population, see Tables S1-S3.
Recruitment	The recruitment of the human participants take place at the University Hospital of Magdeburg. Only subjects who were not taking immunosuppressive drugs, had not taken antibiotics within 4 weeks prior to blood collection, and had no known immunodeficiencies or serious pre-existing conditions were included in the study. so, the study is limited to healthy volunteers. It can therefore only be speculated how the immune system of non-healthy people reacts to the bacteria studied. To fill this gap, adults who were severely affected by COVID-19 were also studied. Here, too, we can only speculate how children, younger people or mildly COVID-19 infected people react to the bacteria studied.
Ethics oversight	The study was reviewed and approved by the Clinical Research Ethics Committee of the University of Magdeburg (certificates 06/11, 79/07, 26/12 and 159/18).

Note that full information on the approval of the study protocol must also be provided in the manuscript.

Field-specific reporting

Please select the one below that is the best fit for your research. If you are not sure, read the appropriate sections before making your selection.

☒ Life sciences ☐ Behavioural & social sciences ☐ Ecological, evolutionary & environmental sciences

For a reference copy of the document with all sections, see nature.com/documents/nr-reporting-summary-flat.pdf

Life sciences study design

All studies must disclose on these points even when the disclosure is negative.

Sample size	For the neonates, children and healthy adults, we used all the samples that were available, as all participants st the time of sample collection and there was s shortage of samples. Our experience and liturature (Vogel et al, 2018) led to the expection that the sample size is sufficient, which was supported by the statistical analyses.
Data exclusions	No data were excluded.
Replication	All experiments were repeated with several different donors in independent experiments. The results were reproducible with a certain deviation.
Randomization	The allocation to the different groups was based on the age of the patients. The age groups were determined in advance of the study. The determination of the age groups was based on previous studies (Knolle et al, 2020; Schmiedeberg et al, 2016).
Blinding	The investigators were only partially blinded to group assignment during data collection and analysis. The examiner did not know to which age group the respondent belonged during data collection and evaluation for the children. For the neonates and adults, this was known. Eine vollständige Verblindung war nicht möglich, da im Vorfeld die Blutproben bei anderen Studienbeteiligten angefragt werden mussten. Ausserdem musste der Prüfer für die spätere Aufarbeitung und Sortierung wissen, um welche Altersgruppe es sich handelt.

Reporting for specific materials, systems and methods

We require information from authors about some types of materials, experimental systems and methods used in many studies. Here, indicate whether each material, system or method listed is relevant to your study. If you are not sure if a list item applies to your research, read the appropriate section before selecting a response.

Materials & experimental systems

n/a	Involved in the study
☐	☒ Antibodies
☒	☐ Eukaryotic cell lines
☒	☐ Palaeontology and archaeology
☒	☐ Animals and other organisms
☒	☐ Clinical data
☒	☐ Dual use research of concern
☒	☐ Plants

Methods

n/a	Involved in the study
☒	☐ ChIP-seq
☐	☒ Flow cytometry
☒	☐ MRI-based neuroimaging

Antibodies

Antibodies used

All antibodies are listed in Table S4, including fluorophores, clones and information on the manufacturer. The dilution of the antibodies ist described in the methods part.

Validation

According to the hompage of Biolegend the following antibodies are verified for human reactivity and tested for application in flow cytometry or intracellular staining for flow cytometry: CD4 –PacificBlue (clone: RPA-T4), CD3 – PerCP (clone: SK7), CD25 – APC-Cy7 (clone:BC96), CD45 – Brilliant Violet 510 (clone: HI30), IL-2 -APC (clone: MQ1-17H12), TNF-α-FITC (clone: Mab11), CD69 – FITC (clone: FN50), anti-T-bet - PE-Cy7 (clone: 4B10), anti-FoxP3 – Alexa Fluor 647(clone: 259D), anti-GATA3 – Alexa Fluor 488 (clone: 16E10A23), HLA-DR – PE-Cy7 (clone:L243), IL-6 – APC (clone:MQ2-13A5) and Annexin V – APC.
According to the hompage of Biolegend the following antibodies are verified for human reactivity and tested for blocking of biological response: LEAF Purified CD11b (clone: ICRF44), LEAF Purified CD18 (clone: TS1/18).
According to the hompage of Biolegend the following antibodies are verified for human reactivity and tested for neutralization of a soluble factor by an antibody: Ultra-LEAF Purified IL-10 (clone: JES3-19F1) and LEAF Purified TGFß (clone: 19D8).
According to the hompage of Biolegend the following antibodies are verified for human reactivity and tested for activation of T-cells: Ultra-LEAF Purified CD3 (clone:UCHT1) and Ultra-LEAF Purified CD28 (clone: CD28.2).
According to the hompage of BD Pharmingen the following antibody is verified for human reactivity and tested for blocking of biological response: CTLA-4 (clone: BNI3).
According to the hompage of BD Biosciences the following antibodies are verified for human reactivity and tested for application in flow cytometry or intracellular staining for flow cytometry: IFN-γ –PE-Cy7 (clone: 4S.B3), RORγt – PE (clone: Q21-559), IL-10 – APC (clone: JES3-19F1).
According to the hompage of Invitrogen the following antibody is verified for human reactivity and tested for application in ntracellular staining for flow cytometry: IL-1ß – PE (clone: CRM56).
According to the hompage of eBioscience the following antibodie is verified for human reactivity and tested for application in ntracellular staining for flow cytometry: IL-17A-PE (clone: eBio64DEC17).
According to the hompage of Miltenyi Biotec the following antibodies are verified for human reactivity and tested for application in flow cytometry: CD14 – VioBlue (clone: REA599), CD16 – APC-Vio770 (clone: REA423), CD45RA – FITC (clone: REA562), CD45RO – PE-Vio770 (clone: REA611), CD4 - FITC (clone: REA613), CD3 - VioGreen (clone: REA623), CD14 – PerCP-Vio700 (clone: REA599).
To test the functionality of the above listed antibodies, all antibodies were controlled against IgG controls, FMO, unstained and resting cells.
For HLA-DR blockade a homemade antibody was used. The used clone L243 is verified for human reactivity as described aon several homepages e.g. Biolegend, Miltenyi Biotec, Thermo Fisher Scientific, Sigma Aldrich, Santa Cruz Biotechnology and Abcam. The andibody was controlled by western blotting and tested in competitive FACS analysis.

Flow Cytometry

Plots

Confirm that:

☒ The axis labels state the marker and fluorochrome used (e.g. CD4-FITC).

☒ The axis scales are clearly visible. Include numbers along axes only for bottom left plot of group (a 'group' is an analysis of identical markers).

☒ All plots are contour plots with outliers or pseudocolor plots.

☒ A numerical value for number of cells or percentage (with statistics) is provided.

Methodology

Sample preparation

Mononuclear cells were obtained from peripheral blood (PB) of healthy donors, surgically excised adenoids of infants suffering from non-inflammatory hypertrophy, or cord blood (CB) by centrifugation of Ficoll-Hypaque gradient. CD14+ monocytes isolated from cord blood, blood of infants, and healthy donors using CD14-MicroBeads (Miltenyi Biotec) and autoMACS-Pro isolation (Miltenyi Biotec) were matured with heat-inactivated (h.i.) Staphylococcus aureus ssp. aureus Rosenbach (ATCC 25923, no toxin producing strain), h.i. Staphylococcus epidermidis (Winslow and Winslow) Evans (ATCC 12228), h.i. or alive Bifidobacterium longum ssp. infantis (ATCC 15697) or SARS-CoV-2-peptide pool PepTivator SARS-CoV-2 Prot_S derived from spike protein or PepTivator SARS-CoV-2 Prot_N derived from N protein (both pools contain lyophilised peptides consisting mainly of 15-mer sequences with 11 amino acid overlap and are used at a concentration of 18.75 ng ml-1, Miltenyi Biotec) over night at 37°C in RPMI 1640 (PAN Biotech) containing 10 % Fetal Bovine Serum (Gibco/Life

	Technologies GmbH); 10 µg ml-1 streptomycin; and 10 U ml-1 penicillin (Life Technologies GmbH). Microbes were washed three times with PBS and centrifuged at 4000xg in between and then killed by heating at 65 °C for 1 h (h.i.) according to standard methods, followed by three freeze-thaw cycles. Protein concentration was determined by the bicinchoninic acid assay (Bio-Rad) according the manufacturer's instructions. The concentration of h.i. bacteria used in co-culture experiments was determined after titration in T-cell proliferation assays using CFSE labelled T-cells, as described below. 5 µg ml-1 of h.i. bacteria S. aureus, 5 µg ml-1 S. epidermidis or 10 µg ml-1 B. infantis gave max. proliferative response and viability of T-cells, when applied to 2.5x105 monocytes per ml for 16 h (Fig. S2B)12. H.i. bacteria were tested for endotoxin concentrations using Pierce LAL Chromogenic Endotoxin Quantitation Kit (Thermo Scientific) and used with less than 0.036 EU ml-1 in bacteria-monocytes co-cultures. Monocytes were then washed twice prior co-culturing with T-cells in a 1:2 (2.5x105 monocytes ml-1 and 5x105 T-cells ml-1). CD4+CD45RA+ T-cells or recent thymic emigrants (CD4+CD45RA+CD31+) were enriched to high purity (>99.6%) by magnetic beads separation with autoMACS-Pro using human naive CD4+ T-cell Isolation Kit or human CD4 + Recent Thymic Emigrant Isolation Kit (Miltenyi Biotec) respectively.<br>A total of 5x105 cells ml-1 enriched CD4+CD45RA+ T-cells or recent thymic emigrants (CD4+CD45RA+CD31+) were stimulated with the bacteria-matured CD14+CD16+ monocytes at a ratio 2:1. For blockage of HLA-DR, anti-HLA-DR (10 µg ml-1, L249, homemade, controlled by Western blotting and competitive FACS analysis) blocking antibody was used. IL-10 was neutralized by using an anti-IL-10 mab (10 µg ml-1, JES3-19F1, Biolegend) and blocking was controlled by LegendPlex (Fig. S7A). For TLR inhibitor experiments, MyD88 inhibitor Pepinh-MYD (50µM, InvivoGen) was used. For blockage of CR3α, anti-CD11b (10 µg ml-1, ICRF44, Biolegend) and anti-CD18 (10 µg ml-1, TS1/18, Biolegend) blocking antibody were used. TGFß blockage was performed by anti-TGFß (10 µg ml-1, 19D8, Biolegend) and blockage of CTLA-4 by anti-CTLA-4 (10 µg ml-1, BNI3, BD Pharmingen). For positive control, T-cells were stimulated routinely with microbeads coated with anti-CD3 (1 µg ml-1; UCHT1) and anti-CD28 (2 µg ml-1; CD28.2) (both Biolegend) at a cell to bead ratio of 2:1.
Instrument	Cytometric analyses were performed using a FACS Conta II (Becton Dickinson) or FACS Fortess X-20 (Becton Dickinson).
Software	FACS Diva software version 3.0.1 (BD Biosciences) was used for the measurements and FlowJo software for the final analysis. Information can also be found in Table S4 and in the mehtods.
Cell population abundance	Naive T-cells are routinely enriched to >99.6% purity from cord blood, blood and adenoids of infnats or children, and adult peripheral blood. The cells were stained with CD4 and gated for leukocytes.
Gating strategy	Gating stragegy can be found in Fig. S2A.

☒ Tick this box to confirm that a figure exemplifying the gating strategy is provided in the Supplementary Information.
